# Design, Synthesis, and Fungicidal Activity of Novel Thiosemicarbazide Derivatives Containing Piperidine Fragments

**DOI:** 10.3390/molecules22122085

**Published:** 2017-12-11

**Authors:** Xuebo Zhang, Peng Lei, Tengda Sun, Xiaoyu Jin, Xinling Yang, Yun Ling

**Affiliations:** Department of Applied Chemistry, College of Science, China Agricultural University, Beijing 100193, China; zhangxueb@cau.edu.cn (X.Z.); leipeng@cau.edu.cn (P.L.); tenda@cau.edu.cn (T.S.); stellajxy@cau.edu.cn (X.J.); yangxl@cau.edu.cn (X.Y.)

**Keywords:** benzaldehyde, thiosemicarbazide, piperidine, synthesis, fungicidal activity

## Abstract

In order to discover novel eco-friendly lead compounds for plant pathogenic fungi control, a series of benzaldehyde thiosemicarbazide derivatives with a piperidine moiety have been designed and synthesized. Fungicidal activities of all the synthesized compounds were evaluated in vitro. The results indicated that all the title compounds exhibited moderate to good fungicidal activities. Compound **3b** displayed excellent activities against *Pythium aphanidermatum*, *Rhizoctonia solani*, *Valsa mali*, and *Gaeu-mannomyces graminsis*, with EC_50_ values lower than 10 μg/mL. Especially, in the case of *Pythium aphanidermatum*, its activity (EC_50_ = 1.6 μg/mL) is superior to the commercial azoxystrobin (EC_50_ = 16.9 μg/mL) and close to fluopicolide (EC_50_ = 1.0 μg/mL). Initial structure–activity relationship (SAR) analysis showed that the heterocyclic piperidine group can influence the biological activities of the title compounds significantly. The fungicidal activity of compounds with piperidine is better than that of compounds without piperidine. The highly-active compound **3b**, with its simple structure and easy synthetic route, is worthy to be further studied as a new lead fungicide.

## 1. Introduction

Plant diseases can significantly affect the yields of crops around the world, with losses up to 25% [1], as well as depreciate the quality and shorten the storage time of the fruits and vegetables [2]. Moreover, some fungi can harm human and animal health due to their mycotoxins [3]. Therefore, fungicide must be used to control fungal plant diseases. However, the extensive use of pesticides has led to residue, resistance, and resurgence, known as “3R” problems. Thus, developing efficient, low-toxicity, environment-friendly pesticides is particularly important. 

**Figure 1:**
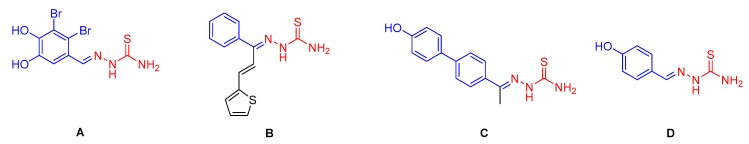
The structure of the benzaldehyde thiosemicarbazide derivatives. (**A**) Anticancer activity; (**B**) antifungal activity; (**C**,**D**) tyrosinase inhibitor.

Natural products, with the characteristics of lower toxicity and less environmental pollution, are often used as lead structures for the discovery of novel green pesticides [4]. Benzaldehyde and their derivatives widely exist in bitter almond oil, walnut oil, orange blossom oil, cinnamon oil, and other natural essential oils. As a spice with an annual demand of about 7000 tons [5], benzaldehyde is widely used in food, beverages, tobacco, and cosmetics. It also play important roles in medical chemistry and agrochemicals due to its derivatives’ (Figure 1) close association with various types of biological properties, such as antibacterial [6], antifungal [7], antioxidant [8], antitrypsnosomal [9], antiviral [10] functions, and so on [7,8]. Particularly, benzaldehyde thiosemicarbazide derivatives have been extensively applied to medicine, materials, pesticides, and other fields thanks to their good bioactivities (Figure 1A–C) [11,12,13,14,15,16,17]. Our team is also devoted to the structure modification of benzaldehyde thiosemicarbazide (Figure 1D) and has found that some analogues showed tyrosinase inhibitor activities in a previous study [18].

**Figure 2:**
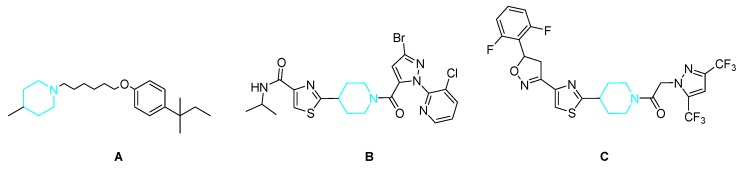
The structure of the piperidine derivatives. (**A**) Histamine H_3_R ligands; (**B**) antifungal activity; (**C**) fungicide oxathiapiprolin.

Heterocyclic compounds have received considerable attention in recent years owing to their broad bioactivities and have become of significant importance in industry and biology [19]. As a saturated heterocyclic, piperidine is a cyclic secondary amine that has been frequently reported for its multiple activities, such as anti-HIV [20], histamine H_3_R ligands (Figure 2A) [21], antimalarial [22], antimicrobial [23], antifungal (Figure 2B) [24], anticoagulant [25], and other biological properties [26]. For instance, oxathiapiprolin (Figure 2C), the first member of the piperidinyl thiazole isoxazoline fungicides developed globally as DuPont™ Zorvec™, can control plant diseases caused by oomycete pathogens in different stages of the pathogen’s life cycle at extremely low concentrations [27].

**Figure 3:**
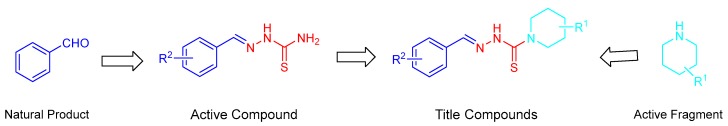
Design strategy of the title compounds thiosemicarbazide derivatives containing piperidine fragments.

In the view of the need of efficient and eco-friendly fungicidal lead compounds, a series of thiosemicarbazone analogues containing heterocyclic a piperidine moiety were designed and synthesized via linking active substructures with the aim to obtain a new prospective lead with a simple structure and good activities. The design strategy of the title compounds are shown in Figure 3. All the compounds were evaluated for their activities against six plant pathogenic fungi. In addition, the initial SAR analysis was also reported in the present work.

## 2. Results and Discussion

### 2.1. Chemistry

**Scheme 1:**
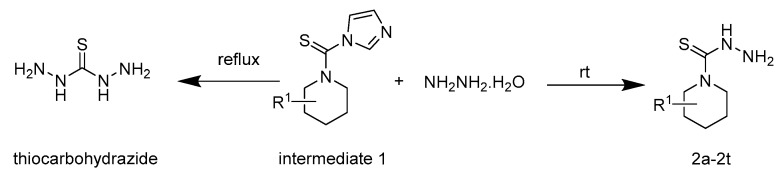
Reaction route of intermediate **1** and hydrazine hydrate at different temperatures.

**Scheme 2:**
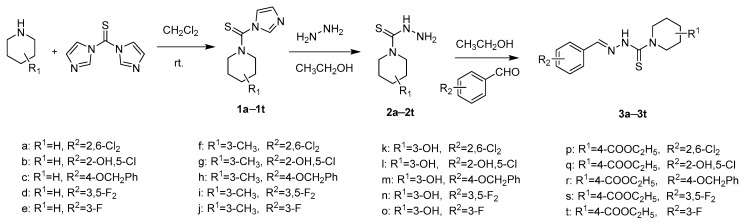
Synthetic route of the title compounds **3a**–**3t**.

The synthetic route for the intermediates and benzaldehyde thiosemicarbazide analogues is illustrated in Scheme 1 and Scheme 2. Intermediate **1** was obtained from substituted piperidine and *N*,*N*′-thiocarbonyldiimidazole via a nucleophilic substitution reaction at room temperature, and then reacted with hydrazine hydrate to afford the key intermediate **2**. The temperature had an important influence on yield of intermediate **2**, and room temperature is favorable for the reaction. C–N bonds connected with imidazole cleavage easily compared to C–N bonds connected with piperidine, intermediate **2** was obtained when intermediate **1** was reacted with hydrazine hydrate under room temperature conditions. Double addition was taken place due to double C–N bonds being activated under reflux conditions. When intermediate **1** was reacted with hydrazine hydrate under reflux conditions, instead of intermediate **2**, thiocarbohydrazide was obtained (Scheme 1).

The title compounds **3a**–**3t** were prepared from intermediate **2** followed by a condensation reaction with substituted benzaldehyde in ethanol as the solvent (Scheme 2).

**Scheme 3:**
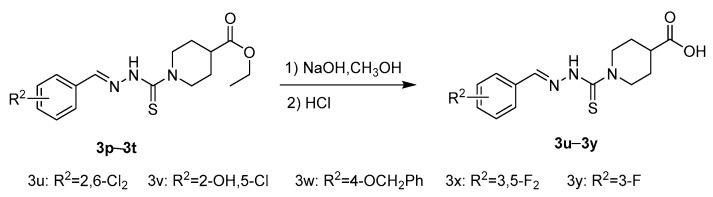
Synthetic route of the title compounds **3u**–**3y**.

Compounds **3u**–**3y** were prepared from compounds **3p**–**3t** through the hydrolysis reaction at room temperature in the NaOH aqueous solution (Scheme 3).

The structures of all synthesized benzaldehyde thiosemicarbazide analogues **3a**–**3y** were confirmed by IR, ^1^H-NMR, ^13^C-NMR, DEPT-135, elemental analysis, or HR-ESI-MS. Their physical and chemical properties and structure characterization were described in Section 3.2. In the IR spectra, the analogues showed strong absorptions around 3300 cm^−1^ due to the N–H stretching vibration. A strong band at about 1700 cm^−1^ was detected because of the C=S stretching vibration. A weak band at about 1650 cm^−1^ was detected because of the C=N stretching vibration. 

In the ^1^H-NMR spectrum of benzaldehyde thiosemicarbazide analogues, a wide single peak in the δ 11.0~11.2 ppm chemical shift range was observed due to the presence of NH protons. The protons of N=CH were observed at δ 8.0~8.2 ppm. The signal of the C–H protons in benzene were clearly observed at δ 7.0~8.0 ppm. The signal of the C–H protons in piperidine were observed at δ 1.0~5.0 ppm. Detailed ^1^H-NMR and ^13^C-NMR spectroscopy characteristics for the compounds are given in the Supporting Information (Figures S3–S6, S8–S53). For further confirmation, DEPT-135 analysis was performed (Figure S7). The signals in the negative direction relative to the original ^13^C-NMR spectrum confirm the existence of CH_2_ groups in the compounds.

### 2.2. Fungicidal Activities

The title compounds were evaluated for their fungicidal activities against six plant pathogenic fungi (*Pythium aphanidermatum*, *Rhizoctonia solani*, *Valsa mali*, *Botrytis cinerea*, *Alternaria solani*, and *Gaeu-mannomyces graminsis*) at the concentration of 50 mg/L as described in Section 3.3. The results were reported in Table 1. Fluopicolide, a commercial fungicide, was used as a positive control. Data listed in Table 1 showed that all the title compounds showed obvious fungicidal activities against all the six tested pathogenic fungi. Some compounds displayed satisfied activities, with inhibition rates against *Pythium aphanidermatum*, *Rhizoctonia solani*, *Valsa mali*, and *Gaeu-mannomyces graminsis* higher than 85%. Notably, compound **3b** was found to display improved fungicidal activities against *Rhizoctonia solani* and *Valsa mali* (93%, 86%, respectively) compared with that of Fluopicolide (43%, 63%, respectively). The inhibitory rates of compound **3b** against *Pythium aphanidermatum* and *Gaeu-mannomyces graminsis* reached 99%, similar to fluopicolide (99% and 95%, respectively).

**Figure 4:**
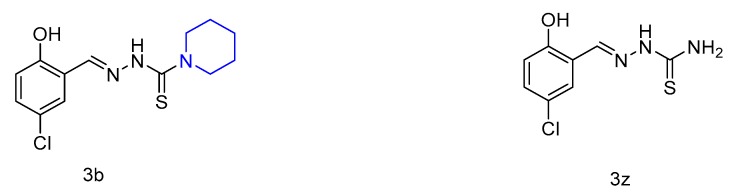
The chemical structure of compounds **3b** and **3z**.

EC_50_ value of some compounds against *Pythium aphanidermatum*, *Rhizoctonia solani*, *Valsa mali*, and *Gaeu-mannomyces graminsis* were further tested (Table 2). In general, for *Rhizoctonia solani*, most compounds showed good antifungal activities with EC_50_ values lower than 10 μg/mL, and compounds **3c** (R^1^ = H, R^2^ = 4-OCH_2_Ph) and **3f** (R^1^ = 3-CH_3_, R^2^ = 2,6-Cl_2_) showed the best activities among all the title compounds with the EC_50_ values of 2.2 μg/mL and 2.1 μg/mL, respectively; for *Pythium aphanidermatum*, compound **3b** (R^1^ = H, R^2^ = 2-OH,5-Cl), and compound **3l** (R^1^ = 3-OH, R^2^ = 2-OH,5-Cl) displayed satisfied activities. Especially, compound **3b** showed excellent activity with the EC_50_ value of 1.6 μg/mL, which is close to fluopicolide (EC_50_ = 1.0 μg/mL), a commercial fungicide to control *Pythium aphanidermatum* pathogens in the market. Structure-activity relationship study showed that: compounds (**3b**, **3g**, **3i**, and **3v**) with 2-OH,5-Cl substituents on phenyl displayed better activities than those compounds with other substituents. In particular, the compound **3b** containing unsubstituted piperidine displayed the most potent fungicidal activities against *Rhizoctonia solani*, *Valsa mali*, *Gaeu-mannomyces graminsis*, and *Pythium aphanidermatum*, with EC_50_ values of 9.6, 2.3, 9.3, and 1.6 μg/mL, respectively. Interestingly, compound **3z** (Figure 4) without piperidine, although with 2-OH, 5-Cl substituents, exhibited much lower fungicidal activities than compound **3b**. This indicated that the piperidine may play an important role on fungicidal activity.

## 3. Materials and Methods

### 3.1. General Information

Melting points of all compounds were determined on an X-4 binocular microscope (Fukai Instrument Co., Beijing, China) without calibration. NMR spectra were recorded on Bruker AM-300 (300 MHz) spectrometer with CDCl_3_ or DMSO-*d*_6_ as the solvent and TMS as the internal standard. Chemical shifts are reported in δ (parts per million) values. Elemental analysis was carried out on a Vario EL III elemental analyzer. High-resolution mass spectra were determined under electron impact (150 eV) conditions using a Bruker APEX IV instrument (Bruker Daltonics, Billerica, MA, USA). All the reagents, substituted piperidines, and benzaldehydes were obtained commercially and used without further purification.

### 3.2. Synthesis of Benzaldehyde Thiosemicarbazide Analogues ***3a**–**3y***

#### 3.2.1. General Procedure for the Preparation of Intermediates **1**

Intermediate **1** was synthesized using the published method [28]. A three-necked round bottom flask was charged with 22 mmol *N,N′*-thiocarbonyldiimidazole in 30 mL CH_2_Cl_2_. To the reaction flask 20 mmol substituted piperidine was added, and the reaction mixture was stirred at room temperature for 3 h. The mixture was washed with 100 mL water (3×). The organic layer was dried over sodium sulfate and the solvent was removed under reduced pressure. The resulting intermediate **1** was directly used in the next step without further purification. Intermediate **1a**: yield 95%, yellow solid, m.p. = 85.0~86.0 °C; ^1^H-NMR (300 MHz, DMSO, Figure S1) δ 7.98 (t, *J* = 1.1 Hz, 1H, imidazole-H), 7.45 (t, *J* = 1.4 Hz, 1H, imidazole-H), 7.05~6.96 (m, 1H, imidazole-H), 4.49~3.27 (m, 4H, piperidine-H), 1.64 (m, 6H, piperidine-H).

#### 3.2.2. General Procedure for the Preparation of Intermediates **2**

On the basis of reported method [28], a three-necked round bottom flask was charged with 19 mmol intermediate **1** in 20 mL anhydrous ethanol. The reaction flask was charged with 19 mmol hydrazine hydrate (80%) via the addition funnel, which was a suspension in 10 mL anhydrous ethanol. The reaction mixture was stirred at room temperature for overnight. After intermediate **1** was completely consumed, the solvent ethanol was evaporated under reduced pressure, and the mixture was filtered and washed with cold ethanol. The resulting intermediate **2** was directly used in the next step without further purification. Intermediate **2a**: yield 76%, white solid, m.p. = 93.0~94.0 °C; ^1^H-NMR (300 MHz, DMSO, Figure S2): 8.87 (s, 1H, NH), 4.66 (s, 2H, NH_2_), 3.70~3.61 (m, 4H, piperidine-H), 1.54 (m, 2H, piperidine-H), 1.42 (m, 4H, piperidine-H).

#### 3.2.3. General Procedure of Benzaldehyde Thiosemicarbazide Analogues **3a**–**3t**

The intermediate **2** (4.7 mmol) was added to a solution of corresponding substituted benzaldehyde (5 mmol) in 15 mL ethanol. Then three drops of glacial acetic acid were added to the reaction mixture as a catalyst. The mixture was stirred at room temperature for 10 min, corresponding precipitate was formed and then reaction 5 h at room temperature. The solid was filtered off and washed with ethanol to give the title compounds **3a**–**3t**. The yields, physicochemical properties and structural characterization data of **3a**–**3t** are as follows:

*(E)-N′′-(2,6-Dichlorobenzylidene)piperidine-1-carbothiohydrazide* (**3a**). White solid, m.p. 153.0~154.0 °C, yield 70%. IR (KBr), ν/cm^−1^: 3188, 1601, 1580, 1236, 777; ^1^H-NMR (DMSO-*d*_6_, 300 MHz) δ: 1.69~1.44 (m, 6H, Piperidine-H), 4.01~3.73 (m, 4H, Piperidine-H), 7.43 (m, 3H, Ar-H), 8.34 (s, 1H, CH=N), 11.22 (s, 1H, NH); ^13^C-NMR (75 MHz, DMSO): 180.43, 138.44, 133.91, 130.92, 130.43, 129.29, 51.55, 25.90, 23.96; Anal. calcd. for C_13_H_15_Cl_2_N_3_S: C 49.37, H 4.78, N 13.29; found C 49.21, H 4.93, N 13.11.

*(E)-N′-(5-Chloro-2-hydroxybenzylidene)piperidine-1-carbothiohydrazide* (**3b**). Yellow solid, m.p. 174.0~175.0 °C, yield 52%. IR (KBr), ν/cm^−1^: 3352, 3003, 1614, 1535, 244, 829; ^1^H-NMR (DMSO-*d*_6_, 300 MHz) δ: 1.59 (m, 6H, Piperidine-H), 3.99~3.78 (m, 4H, Piperidine-H), 6.90 (d, *J* = 8.8 Hz, 1H, Ar-H), 7.26 (dd, *J* = 8.8, 2.6 Hz, 1H, Ar-H), 7.51 (d, *J* = 2.6 Hz, 1H, Ar-H), 8.39 (s, 1H, CH=N), 11.48 (s, 1H, NH), 11.68 (s, 1H, Ar-OH); ^13^C-NMR (75 MHz, DMSO): 178.88, 155.87, 143.99, 130.20, 128.64, 122.67, 120.40, 118.45, 49.91, 25.66, 23.96; Anal. calcd. for C_13_H_16_ClN_3_OS: C 52.43, H 5.42, N 14.11; found C 52.31, H 5.20, N 13.99.

*(E)-N′-(4-(Benzyloxy)benzylidene)piperidine-1-carbothiohydrazide* (**3c**). Yellow solid, m.p. 120.0~121.0 °C, yield 66%. IR (KBr), ν/cm^−1^: 3151, 3033, 1603, 1506, 1248, 835; ^1^H-NMR (DMSO-*d*_6_, 300 MHz) δ: 1.61 (s, 6H, Piperidine-H), 3.83 (s, 4H, Piperidine-H), 5.13 (s, 2H, Ar–CH_2_–O–), 7.05 (m, 2H, Ar-H), 7.61~7.25 (m, 7H, Ar-H), 8.05 (s, 1H, CH=N), 10.87 (s, 1H, NH); ^13^C-NMR (75 MHz, DMSO): 180.25, 159.71, 143.22, 136.90, 128.57, 128.34, 128.02, 127.84, 127.37, 115.34, 69.49, 51.37, 25.92, 24.07; Anal. calcd. for C_20_H_23_N_3_OS: C 67.96, H 6.56, N 11.89; found C 68.07, H 6.58, N 11.72.

*(E)-N′-(3,5-Difluorobenzylidene)piperidine-1-carbothiohydrazide* (**3d**). Yellow solid, m.p. 113.0~114.7 °C, yield 78%. IR (KBr), ν/cm^−1^: 3148, 3057, 1623, 1602, 1495, 1248, 858; ^1^H-NMR (DMSO-*d*_6_, 300 MHz) δ: 1.62 (s, 6H, Piperidine-H), 3.85 (m, 4H, Piperidine-H), 7.39~7.15 (m, 3H, Ar-H), 8.08 (s, 1H, CH=N), 11.21 (s, 1H, NH); ^13^C-NMR (75 MHz, DMSO): 180.37, 164.40, 161.14, 140.54, 138.53, 109.61, 109.27, 105.02, 104.68, 104.33, 51.30, 25.87, 24.00; Anal. calcd. for C_13_H_15_F_2_N_3_S: C 55.11, H 5.34, N 14.83; found C 54.89, H 5.10, N 14.93.

*(E)-N′-(3-Fluorobenzylidene)piperidine-1-carbothiohydrazide* (**3e**). White solid, m.p. 108.0~109.0 °C, yield 54%. IR (KBr), ν/cm^−1^: 3175, 3075, 1603, 1576, 1507, 1244, 7813; ^1^H-NMR (DMSO-*d*_6_, 300 MHz) δ: 1.62 (s, 6H, Piperidine-H), 3.85 (m, 4H, Piperidine-H), 7.21 (m, 1H, Ar-H), 7.52~7.36 (m, 3H, Ar-H), 8.10 (s, 1H, CH=N), 11.09 (s, 1H, NH); ^13^C-NMR (75 MHz, DMSO): 180.37 (s), 164.17 (s), 160.94 (s), 141.79 (d), 137.25 (d), 131.00 (d), 123.23 (d), 116.51 (s), 116.23 (s), 112.70 (s), 112.41 (s), 51.34 (s), 25.90 (s), 24.03 (s); HRMS (ESI^+^) *m*/*z* calcd for C_13_H_16_FN_3_S, 266.1122 [M], found 266.1122.

*(E)-N′-(2,6-Dichlorobenzylidene)-3-methylpiperidine-1-carbothiohydrazide* (**3f**). White solid, m.p. 104.9~105.9 °C, yield 48%. IR (KBr), ν/cm^−1^: 3153, 3047, 1580, 1557, 1505, 1243, 782; ^1^H-NMR (DMSO-*d*_6_, 300 MHz) δ: 0.94 (t, *J* = 6.0 Hz, 3H, CH_3_), 1.30~1.11 (m, 1H, Piperidine-CH), 1.94~1.61 (m, 4H, Piperidine-H), 2.95~2.74 (m, 1H, Piperidine-H), 3.21~3.09( m, 1H, Piperidine-H), 4.84~4.58 (m,2H, Piperidine-H), 7.46~7.20 (m, 3H, Ar-H), 7.90 (s, 1H, CH=N), δ 8.81 (s, 1H, NH); ^13^C-NMR (75 MHz, DMSO): 180.43, 138.48, 133.90, 130.97, 130.49, 129.28, 57.45, 51.06, 32.45, 31.19, 25.08, 18.77; Anal. calcd. for C_14_H_17_Cl_2_N_3_S: C 50.91, H 5.19, N 12.72; found C 51.05, H 5.36, N 12.93.

*(E)-N′-(5-Chloro-2-hydroxybenzylidene)-3-methylpiperidine-1-carbothiohydrazide* (**3g**). Yellow solid, m.p. 187.0~188.0 °C, yield 88%. IR (KBr), ν/cm^−1^: 3692, 1616, 1539, 1490, 1248, 837; ^1^H-NMR (DMSO-*d*_6_, 300 MHz) δ: 0.97 (d, *J* = 6.6 Hz, 3H, CH_3_), 1.33~1.14 (m, 1H, Piperidine-CH), 1.99~1.58 (m, 4H, Piperidine-H), 2.83 (m, 1H, Piperidine-H), 3.27~3.07 (m, 1H, Piperidine-H), 4.52 (m, 2H, Piperidine-H), 6.93 (m, 1H, Ar-H), 7.27~7.13 (m, 2H, Ar-H), 7.86 (s, 1H, CH=N), 8.62 (s, 1H, NH),10.49 (s, 1H, Ar-OH); ^13^C-NMR (75 MHz, DMSO): 178.88, 155.86, 143.91, 130.22, 128.57, 122.68, 120.42, 118.45, 55.85, 49.45, 32.48, 31.12, 24.97, 18.78; Anal. calcd. for C_14_H_18_ClN_3_OS: C 53.93, H 5.82, N 13.48; found C 53.80, H 5.71, N 13.39.

*(E)-N′-(4-(Benzyloxy)benzylidene)-3-methylpiperidine-1-carbothiohydrazide* (**3h**). Yellow solid, m.p. 122.0~123.4 °C, yield 60%. IR (KBr), ν/cm^−1^: 3160, 3032, 1606, 1507, 1243, 827; ^1^H-NMR (DMSO-*d*_6_, 300 MHz) δ: 0.86 (d, *J* = 6.6 Hz, 3H, CH_3_), 1.17 (m, 1H, Piperidine-H), 1.58~1.42 (m, 1H, Piperidine-H), 1.74~1.59 (m, 2H, Piperidine-H), 1.79 (m, 1H, Piperidine-H), 2.76 (m, 1H, Piperidine-H), 3.04 (m, 1H, Piperidine-H), 4.66~4.37 (m, 2H, Piperidine-H), 5.13 (s, 2H, Ar-CH_2_-O), 7.06 (m, 2H, Ar-H), 7.59~7.28 (m, 7H, Ar-H), 8.05 (s, 1H, CH=N), 10.88 (s, 1H, NH); ^13^C-NMR (75 MHz, DMSO): 180.13, 159.72, 143.28, 136.90, 128.57, 128.31, 128.02, 127.84, 127.37, 115.34, 69.49, 57.35, 50.88, 32.59, 31.28, 25.21, 18.81; HRMS (ESI^+^) *m*/*z* calcd for C_21_H_25_N_3_OS, 368.1791 (M), found 368.1794.

*(E)-N′-(3,5-Difluorobenzylidene)-3-methylpiperidine-1-carbothiohydrazide* (**3i**). Yellow solid, m.p. 111.0~112.0 °C, yield 22%. IR (KBr), ν/cm^−1^: 3155, 3096, 1619, 1507, 1247, 852; ^1^H-NMR (DMSO-*d*_6_, 300 MHz) δ: 0.87 (d, *J* = 6.6 Hz, 3H, CH_3_), 1.28~1.10 (m, 1H, Piperidine-H), 1.79~1.50 (m, 4H, Piperidine-H), 3.15~2.80 (m, 2H, Piperidine-H), 4.51 (m, 2H, Piperidine-H), 7.38~7.17 (m, 3H, Ar-H), 8.08 (s, 1H, CH=N), 11.23 (s, 1H, NH); ^13^C-NMR (75 MHz, DMSO): 180.26 (s), 164.40 (d), 161.14 (d), 140.53 (s), 138.59 (t), 109.41 (d), 104.6 (t), 57.24 (s), 50.85 (s), 32.52 (s), 31.30 (s), 25.17 (s), 18.70 (s); Anal. calcd. for C_14_H_17_F_2_N_3_OS: C 56.55, H 5.76, N 14.13; found C 56.69, H 5.70, N 13.97.°C

*(E)-N′-(3-Fluorobenzylidene)-3-methylpiperidine-1-carbothiohydrazide* (**3j**). Yellow solid, m.p. 81.0~82.0 °C, yield 21%. IR (KBr), ν/cm^−1^: 3155, 3096, 1619, 1507, 1247, 852; ^1^H-NMR (DMSO-*d*_6_, 300 MHz) δ: 0.87 (d, *J* = 6.5 Hz, 3H, CH_3_), 1.18 (m, 1H, Piperidine-H), 1.88~1.41 (m, 4H, Piperidine-H), 3.08~2.70 (m, 2H, Piperidine-H), 4.52 (m, 2H, Piperidine-H), 7.52~7.15 (m, 4H, Ar-H), 8.11 (s, 1H, CH=N), 11.10 (s, 1H, NH); ^13^C-NMR (75 MHz, DMSO): 180.28 (s), 164.18 (s), 160.95 (s), 141.81 (d), 137.26 (d), 131.00 (d), 123.23 (d), 116.52 (s), 116.23 (s), 112.63 (s), 112.33 (s), 57.30 (s), 50.89 (s), 32.54 (s), 31.30 (s), 25.19 (s), 18.74 (s); Anal. calcd. for C_14_H_18_FN_3_S: C 60.19, H 6.49, N 15.04; found C 60.03, H 6.20, N 14.93.

*(E)-N′-(2,6-Dichlorobenzylidene)-3-hydroxypiperidine-1-carbothiohydrazide* (**3k**). White solid, m.p. 164.8~165.9 °C, yield 37%. IR (KBr), ν/cm^−1^: 3329, 3217, 3049, 1581, 1544, 1511, 1252, 774; ^1^H-NMR (DMSO-*d*_6_, 300 MHz) δ: 1.41 (m, 2H, Piperidine-H), 1.99~1.61 (m, 2H, Piperidine-H), 3.09 (m, 1H, Piperidine-H), 3.52~3.26 (m, 2H, Piperidine-H), 4.38 (m, 2H, Piperidine-H), 4.92 (d, *J* = 4.4 Hz, 1H, Piperidine-OH), 7.44 (m, 3H, Ar-H), 8.36 (s, 1H, CH=N), 11.26 (s, 1H, NH); ^13^C-NMR (75 MHz, DMSO): 180.96, 138.67, 133.94, 130.94, 130.36, 129.30, 65.43, 57.15, 50.59, 33.01, 22.94; Anal. calcd. for C_13_H_15_Cl_2_N_3_OS: C 47.00, H 4.55, N 12.65; found C 47.02, H 4.65, N 12.43.

*(E)-N′-(5-Chloro-2-hydroxybenzylidene)-3-hydroxypiperidine-1-carbothiohydrazide* (**3l**). White solid, m.p. 185.8~186.5 °C, yield 27%. IR (KBr), ν/cm^−1^: 3215, 3007, 1618, 1600, 1563, 1511, 1249, 817; ^1^H-NMR (DMSO-*d*_6_, 300 MHz) δ: 1.43 (m, 2H, Piperidine-H), 1.81 (m, 2H, Piperidine-H), 3.16 (m, 1H, Piperidine-H), 3.53 (s, 2H, Piperidine-H), 4.23 (m, 1H, Piperidine-H), 4.44 (m, 1H, Piperidine-H), 4.98 (s, 1H, Piperidine-OH), 6.90 (d, *J* = 8.7 Hz, 1H, Ar-H), 7.26 (dd, *J* = 8.7, 2.5 Hz, 1H, Ar-H), 7.50 (d, *J* = 2.5 Hz, 1H, Ar-H), 8.39 (s, 1H, CH=N), 11.50 (s, 1H, NH), 11.68 (s, 1H, Ar-OH); ^13^C-NMR (75 MHz, DMSO): 179.46, 155.87, 144.10, 130.24, 128.70, 122.68, 120.35, 118.45, 65.24, 55.53, 49.10, 32.95, 22.66; Anal. calcd. for C_13_H_16_ClN_3_O_2_S: C 49.76, H 5.14, N 13.39; found C 50.01, H 5.31, N 13.34.

*(E)-N′-(4-(Benzyloxy)benzylidene)-3-hydroxypiperidine-1-carbothiohydrazide* (**3m**). Yellow solid, m.p. 152.6~153.7 °C, yield 46%. IR (KBr), ν/cm^−1^: 3315, 3177, 3008, 1602, 1511, 1259, 832; ^1^H-NMR (DMSO-*d*_6_, 300 MHz) δ: 1.43 (m, 2H, Piperidine-H), 1.82 (m, 2H, Piperidine-H), 3.03 (m, 1H, Piperidine-H), 3.26~3.16 (m, 1H, Piperidine-H), 3.55 (m, 1H, Piperidine-H), 4.38 (m, 2H, Piperidine-H), 4.96 (d, *J* = 4.3 Hz, 1H, Piperidine-OH), 5.18 (s, 2H, Ar-CH_2_-O), 7.61~7.01 (m, 9H, Ar-H), 8.05 (s, 1H, CH=N), 10.92 (s, 1H, NH); ^13^C-NMR (75 MHz, DMSO): 180.61, 159.74, 143.39, 136.91, 128.58, 128.43, 128.03, 127.84, 127.32, 115.34, 69.49, 65.59, 57.07, 50.52, 33.19, 23.11; Anal. calcd. for C_20_H_23_N_3_O_2_S: C 65.02, H 6.27, N 11.37; found C 64.97, H 6.37, N 11.09.

*(E)-N′-(3,5-Difluorobenzylidene)-3-hydroxypiperidine-1-carbothiohydrazide* (**3n**). Yellow solid, m.p. 145.8~146.6 °C, yield 23%. IR (KBr), ν/cm^−1^: 3254, 3090, 3026, 1605, 1548, 1236, 851; ^1^H-NMR (DMSO-*d*_6_, 300 MHz) δ: 1.44 (m, 2H, Piperidine-H), 1.88 (m, 2H, Piperidine-H), 3.10 (m, 1H, Piperidine-H), 3.26 (s, 1H, Piperidine-H), 3.56 (d, *J* = 4.1 Hz, 1H, Piperidine-H), 4.33 (m, 2H, Piperidine-H), 4.95 (d, *J* = 4.3 Hz, 1H, Piperidine-OH), 7.51~6.99 (m, 3H, Ar-H), 8.08 (s, 1H, CH=N), 11.25 (s, 1H, NH); ^13^C-NMR (75 MHz, DMSO): 180.79 (s), 164.39 (d), 161.13 (d), 140.66 (s), 138.53 (t), 109.50 (q), 104.6 (t), 65.55 (s), 56.93 (s), 50.52 (s), 33.03 (s), 22.93 (s); Anal. calcd. for C_13_H_15_F_2_N_3_OS: C 52.16, H 5.05, N 14.04; found C 52.20, H 5.13, N 13.98.

*(E)-N′-(3-Fluorobenzylidene)-3-hydroxypiperidine-1-carbothiohydrazide* (**3o**). Yellow solid, m.p. 127.0~128.0 °C, yield 34%. IR (KBr), ν/cm^−1^: 3064, 1605, 1558, 795; ^1^H-NMR (DMSO-*d*_6_, 300 MHz) δ: 1.62~1.27 (m, 2H, Piperidine-H), 1.83 (m, 2H, Piperidine-H), 3.09 (m, 1H, Piperidine-H), 3.56~3.26 (m, 2H, Piperidine-H), 4.36 (m, 2H, Piperidine-H), 4.95 (d, *J* = 3.9 Hz, 1H, Piperidine-OH), 7.30~7.12 (m, 1H, Ar-H), 7.55~7.35 (m, 3H, Ar-H), 8.11 (s, 1H, CH=N), 11.13 (s, 1H, NH); ^13^C-NMR (75 MHz, DMSO): 180.79 (s), 164.18 (s), 160.94 (s), 141.95 (d), 137.20 (d), 130.99 (d), 123.33 (d), 116.56 (s), 116.27 (s), 112.76 (s), 112.46 (s), 65.56 (s), 56.99 (s), 50.53 (s), 33.09 (s), 22.99 (s); HRMS (ESI^+^) *m*/*z* calcd for C_13_H_16_FN_3_OS, 282.1071 [M], found 282.1069.

*Ethyl (E)-1-(2-(2,6-dichlorobenzylidene)hydrazine-1-carbonothioyl)piperidine-4-carboxylate* (**3p**). White solid, m.p. 122.3~123.4 °C, yield 75%. IR (KBr), ν/cm^−1^: 3166, 3046, 1729, 1667, 1582, 1270; ^1^H-NMR (DMSO-*d*_6_, 300 MHz) δ: 1.17 (t, *J* = 7.1 Hz, 3H, CH_3_), 1.70~1.49 (m, 2H, Piperidine-H), 1.87 (m, 2H, Piperidine-H), 2.76~2.59 (m, 1H, Piperidine-H), 3.30~3.20 (m, 2H, Piperidine-H), 4.06 (q, *J* = 7.1 Hz, 2H, CH_2_CH_3_), 4.55 (m, 2H, Piperidine-H), 7.40 (m, 1H, Ar-H), 7.54 (m, 2H, Ar-H), 8.36 (s, 1H, CH=N), 11.33 (s, 1H, NH); ^13^C-NMR (101 MHz, DMSO): 181.18, 174.25, 139.16, 134.26, 131.38, 130.67, 129.67, 60.47, 50.04, 28.46, 14.53; Anal. calcd. for C_16_H_19_Cl_2_N_3_O_2_S: C 49.49, H 4.93, N 10.82; found C 49.20, H 4.72, N 10.56.

*Ethyl (E)-1-(2-(5-chloro-2-hydroxybenzylidene)hydrazine-1-carbonothioyl)piperidine-4-carboxylate* (**3q**). Yellow solid, m.p. 184.6~185.4 °C, yield 88%. IR (KBr), ν/cm^−1^: 3135, 3039, 1731, 1616, 1548, 1266, 822; ^1^H-NMR (DMSO-*d*_6_, 300 MHz) δ: 1.18 (t, *J* = 7.1 Hz, 3H, CH_3_), 1.58 (m, 2H, Piperidine-H), 1.91 (m, 2H, Piperidine-H), 2.82~2.59 (m, 1H, Piperidine-H), 3.28 (m, 2H, Piperidine-H), 4.08 (q, *J* = 7.1 Hz, 2H, CH_2_CH_3_), 4.56 (m, 2H, Piperidine-H), 6.91 (d, *J* = 8.8 Hz, 1H, Ar-H), 7.27 (dd, *J* = 8.8, 2.7 Hz, 1H, Ar-H), 7.53 (d, *J* = 2.7 Hz, 1H, Ar-H), 8.40 (s, 1H, CH=N), 11.54 (s, 1H, NH), 11.63 (s, 1H, Ar-OH); ^13^C-NMR (101 MHz, DMSO): 179.64, 174.17, 156.22, 144.55, 130.66, 128.94, 123.06, 120.72, 118.80, 60.51, 48.45, 28.20, 14.54; Anal. calcd. for C_16_H_20_ClN_3_O_3_S: C 51.96, H 5.45, N 11.36; found C 52.20, H 5.54, N 11.43.

*Ethyl (E)-1-(2-(4-(benzyloxy)benzylidene)hydrazine-1-carbonothioyl)piperidine-4-carboxylate* (**3r**). Yellow solid, m.p. 118.8~119.6 °C, yield 91%. IR (KBr), ν/cm^−1^: 3167, 3062, 1731, 1599, 1504, 1242, 829; ^1^H-NMR (DMSO-*d*_6_, 300 MHz) δ: 1.19 (t, *J* = 7.1 Hz, 3H, CH_3_), 1.63 (m, 2H, Piperidine-H), 1.90 (m, 2H, Piperidine-H), 2.78~2.58 (m, 1H, Piperidine-H), 3.25 (m, 2H, Piperidine-H), 4.08 (q, *J* = 7.1 Hz, 2H, CH_2_CH_3_), 4.51 (m, 2H, Piperidine-H), 5.14 (s, 2H, Ar-CH_2_-O), 7.06 (d, *J* = 8.8 Hz, 2H, Ar-H), 7.49~7.27 (m, 5H, Ar-H), 7.55 (d, *J* = 8.8 Hz, 2H, Ar-H), 8.06 (s, 1H, CH=N),10.99 (s, 1H, NH); ^13^C-NMR (101 MHz, DMSO): 180.89, 174.33, 160.14, 143.99, 137.24, 128.92, 128.75, 128.37, 128.19, 127.59, 115.70, 69.83, 60.48, 49.80, 28.45, 14.55; Anal. calcd. for C_23_H_27_N_3_O_3_S: C 64.92, H 6.40, N 9.87; found C 64.99, H 6.34, N 9.88.

Ethyl *(E)-1-(2-(3,5-difluorobenzylidene)hydrazine-1-carbonothioyl)piperidine-4-carboxylate* (**3s**). White solid, m.p. 127.2~128.3 °C, yield 89%. IR (KBr), ν/cm^−1^: 3139, 3059, 1728, 1609, 1539, 1515, 1273, 859; ^1^H-NMR (DMSO-*d*_6_, 300 MHz) δ: 1.18 (t, *J* = 7.1 Hz, 3H, CH_3_), 1.63 (m, 2H, Piperidine-H), 1.92 (m, 2H, Piperidine-H), 2.88~2.58 (m, 1H, Piperidine-H), 3.26 (s, 2H, Piperidine-H), 4.08 (q, *J* = 7.1 Hz, 2H, CH_2_CH_3_), 4.50 (m, 2H, Piperidine-H), 7.49~7.03 (m, 3H, Ar-H), 8.09 (s, 1H, CH=N), 11.32 (s, 1H, NH); ^13^C-NMR (101 MHz, DMSO): 181.11 (s), 174.23 (s), 164.33 (d), 161.89 (d), 141.24 (s), 138.83 (t), 110.18~109.55 (q), 105.13 (t), 60.48 (s), 49.75 (s), 28.41 (s), 14.50 (s); Anal. calcd. for C_16_H_19_F_2_N_3_O_2_S: C 54.07, H 5.39, N 11.82; found C 54.35, H 5.34, N 11.81.

Ethyl *(E)-1-(2-(3-fluorobenzylidene)hydrazine-1-carbonothioyl)piperidine-4-carboxylate* (**3t**). White solid, m.p. 130.0~131.0 °C, yield 78%. IR (KBr), ν/cm^−1^: 3139, 3055, 1728, 1608, 1515, 782; ^1^H-NMR (DMSO-*d*_6_, 300 MHz) δ: 1.18 (t, *J* = 7.1 Hz, 3H, CH_3_),1.71~1.53 (m, 2H, Piperidine-H), 1.91 (m, 2H, Piperidine-H), 2.69 (m, 1H, Piperidine-H), 3.3 (m, 2H, Piperidine-H), 4.08 (q, *J* = 7.1 Hz, 2H, CH_2_CH_3_), 4.50 (m, 2H, Piperidine-H), 7.29~7.17 (m, 1H, Ar-H), 7.60~7.33 (m, 3H, Ar-H), 8.12 (s, 1H, CH=N), 11.20 (s, 1H, NH); ^13^C-NMR (101 MHz, DMSO): 181.09 (s), 174.26 (s), 164.11 (s), 161.69 (s), 142.54 (d), 137.49 (d), 131.37 (d), 123.66 (d), 116.80 (d), 112.92 (d), 60.48 (s), 49.78 (s), 28.43 (s), 14.52 (s); Anal. calcd. for C_16_H_20_F_2_N_3_O_2_S: C 56.96, H 5.97, N 12.45; found C 57.23, H 5.99, N 12.62.

#### 3.2.4. General Procedure of Benzaldehyde Thiosemicarbazide Analoges **3u**–**3y**

A three-necked round bottom flask was charged with compounds **3p**–**3t** (8 mmol), 30 mL ethanol aqueous solution (V_ethanol_:V_water_ = 2:1) was then added. After 16 mL 1.5 mol/L NaOH aqueous solution was added, the reaction mixture was stirred at room temperature for 2 h. TLC monitored the progress of the reaction, and after the ester (**3p**–**3t**) reaction completely, the reaction mixture was cooled to 0 °C, and concentrated HCl was added slowly until PH = 1. The solid was filtered off and washed with water to give the title compounds **3u**–**3y**.

*(E)-1-(2-(2,6-Dichlorobenzylidene)hydrazine-1-carbonothioyl)piperidine-4-carboxylic acid* (**3u**). White solid, m.p. 169.9~170.6 °C, yield 99.8%. IR (KBr), ν/cm^−1^: 3195, 3013, 1693, 1608, 1512, 778; ^1^H-NMR (DMSO-*d*_6_, 300 MHz) δ: 1.57 (m, 2H, Piperidine-H), 1.86 (m, 2H, Piperidine-H), 2.56 (m, 1H, Piperidine-H), 3.25 (d, *J* = 11.0 Hz, 2H, Piperidine-H), 4.55 (m, 2H, Piperidine-H), 7.44 (m, 3H, Ar-H), 8.39 (s, 1H, CH=N), 11.37 (s, 1H, NH), 12.21 (s, 1H, COOH); ^13^C-NMR (101 MHz, DMSO): 181.07, 175.97, 139.30, 134.25, 131.33, 130.77, 129.64, 50.09, 40.35, 28.56; HRMS (ESI^+^) *m*/*z* calcd. for C_14_H_15_Cl_2_N_3_O_2_S, 360.0335 (M); found 360.0331.

*(E)-1-(2-(5-Chloro-2-hydroxybenzylidene)hydrazine-1-carbonothioyl)piperidine-4-carboxylic acid* (**3v**). Yellow solid, m.p. 197.4~198.1 °C, yield 99.3%. IR (KBr), ν/cm^−1^: 3471, 3352, 3289, 1692, 1610, 1537, 1261, 834; ^1^H-NMR (DMSO-*d*_6_, 300 MHz) δ: 1.57 (m, 2H, Piperidine-H), 1.90 (m, 2H, Piperidine-H), 2.69~2.53 (m, 1H, Piperidine-H), 3.28 (m, 2H, Piperidine-H), 4.56 (m, 2H, Piperidine-H), 6.91 (d, *J* = 8.8 Hz, 1H, Ar-H), 7.27 (dd, *J* = 8.8, 2.7 Hz, 1H, Ar-H), 7.52 (d, *J* = 2.7 Hz, 1H, Ar-H), 8.42 (s, 1H, CH=N), 11.66 (s, 3H, NH, OH, COOH); ^13^C-NMR (101 MHz, DMSO): 179.58, 175.90, 156.24, 144.62, 130.61, 128.98, 123.03, 120.70, 118.81, 48.59, 40.31, 28.29; Anal. calcd. for C_16_H_16_ClN_3_O_3_S: C 49.20, H 4.72, N 12.29; found C 48.91, H 4.79, N 12.05.

*(E)-1-(2-(4-(Benzyloxy)benzylidene)hydrazine-1-carbonothioyl)piperidine-4-carboxylic acid* (**3w**). Yellow solid, m.p. 165.8~166.8 °C, yield 96%. IR (KBr), ν/cm^−1^: 3229, 3034, 1701, 1602, 1572, 1510, 1239, 840; ^1^H-NMR (DMSO-*d*_6_, 300 MHz) δ: 1.61 (m, 2H, Piperidine-H), 1.90 (m, 2H, Piperidine-H), 2.58 (m, 1H, Piperidine-H), 3.24 (m, 2H, Piperidine-H), 4.51 (m, 2H, Piperidine-H), 5.14 (s, 2H, Ar-CH_2_-O), 7.07 (d, *J* = 8.6 Hz, 2H, Ar-H), 7.48~7.28 (m, 5H, Ar-H), 7.55 (d, *J* = 8.6 Hz, 2H, Ar-H), 8.07 (s, 1H, CH=N), 10.98 (s, 1H, NH), 12.28 (s, 1H, COOH); ^13^C-NMR (101 MHz, DMSO): 180.79, 176.23, 160.12, 143.94, 137.24, 128.92, 128.74, 128.38, 128.20, 127.62, 115.71, 69.83, 49.99, 40.63, 28.62; Anal. calcd. for C_21_H_23_N_3_O_3_S: C 63.46, H 5.83, N 10.57; found C 63.24, H 6.00, N 10.38.

*(E)-1-(2-(3,5-Difluorobenzylidene)hydrazine-1-carbonothioyl)piperidine-4-carboxylic acid* (**3x**). White solid, m.p. 159.4~160.0 °C, yield 85%. IR (KBr), ν/cm^−1^: 3161, 3080, 1702, 1620, 1585, 1505, 1203, 860; ^1^H-NMR (DMSO-*d*_6_, 300 MHz) δ: 1.60 (m, 2H, Piperidine-H), 1.90 (m, 2H, Piperidine-H), 2.59 (m, 1H, Piperidine-H), 3.30 (m, 2H, Piperidine-H), 4.49 (m, 2H, Piperidine-H), 7.51~7.09 (m, 3H, Ar-H), 8.09 (s, 1H, CH=N), 11.31 (s, 1H, NH), 12.30 (s, 1H, COOH); ^13^C-NMR (101 MHz, DMSO): 181.00 (s), 175.97 (s), 164.33 (d), 161.88 (d), 141.26 (s), 138.86 (t), 110.13~109.53 (q), 105.10 (t), 49.87 (s), 40.40 (s), 28.49 (s); Anal. calcd. for C_14_H_15_F_2_N_3_O_2_S: C 51.37, H 4.62, N 12.84; found C 51.10, H 4.42, N 12.59.

*(E)-1-(2-(3-fluorobenzylidene)hydrazine-1-carbonothioyl)piperidine-4-carboxylic acid* (**3y**). White solid, m.p. 165.2~165.9 °C, yield 87%. IR (KBr), ν/cm^−1^: 3155, 3080, 1708, 1608, 1515, 782; ^1^H-NMR (DMSO-*d*_6_, 300 MHz) δ: 1.61 (m, 2H, Piperidine-H), 1.90 (m, 2H, Piperidine-H), 2.59 (m, 1H, Piperidine-H), 3.35 (s, 2H, Piperidine-H), 4.50 (m, 2H, Piperidine-H), 7.67~7.04 (m, 4H, Ar-H), 8.11 (s, 1H, CH=N), 11.19 (s, 1H, NH), 12.32 (s, 1H, COOH); ^13^C-NMR (101 MHz, DMSO): 181.00 (s), 176.00 (s), 164.10 (s), 161.68 (s), 142.51 (d), 137.50 (d), 131.36 (d), 123.62 (d), 116.80 (d), 113.00 (d), 49.94 (s), 40.41 (s), 28.51 (s); Anal. calcd. for C_14_H_16_FN_3_O_2_S: C 54.36, H 5.21, N 13.58; found C 54.35, H 5.43, N 13.49.

### 3.3. Biological Assay

All the title compounds were evaluated for their in vitro fungicidal activities against six pathogenic fungi, using the mycelium growth rate method according to the literature [29]. Fungi tested in this article included *Pythium aphanidermatum*, *Rhizoctonia solani*, *Valsa mali*, *Botrytis cinerea*, *Alternaria solani*, and *Gaeu-mannomyces graminsis*. The tested compounds were weighted 10 mg and dissolved in 1 mL DMSO, prepared into 10,000 μg/mL concentration solution. Then it was mixed well with 199 mL PDA (potato dextrose agar). The medium containing compounds at a concentration of 50 mg/L for the initial screening was poured into sterilized Petri dishes (d = 90 cm), each group of three parallel tests. After the dishes were cooled, the mycelia disks of 7 mm diameter were inoculated in the center of the Petri dishes and incubated at 25 °C for 2–7 days. The DMSO without sample was used as blank control. The hypha diameter was measured by cross bracketing method. The commercial fungicides fluopicolide, azoxystrobin, and pyraclostrobin were used as positive controls. Fluopicolide is the main pesticide to control *Pythium aphanidermatum* pathogens on the market, and azoxystrobin and pyraclostrobin are the first and second largest fungicides on the market, respectively. The inhibition rate of the title compounds on the fungi was calculated by the following formula:

Inhibition rate (%) = [(C-T)/(C-7 mm)] × 100%

where C represents the average diameter of fungal growth on untreated PDA, and T represents the average diameter of fungi on treated PDA.

## 4. Conclusions

A series of novel benzaldehyde thiosemicarbazide derivatives containing heterocycle piperidine were designed and synthesized using the linking active substructure method. The in vitro bioassay results indicated that all the title compounds exhibited obvious antifungal activities against six pathogenic fungi, especially against *Pythium aphanidermatum*, *Rhizoctonia solani*, *Valsa mali*, and *Gaeu-mannomyces graminsis.* Compound **3b** showed satisfying activity against four tested pathogenic fungi with EC_50_ values lower than 10 μg/mL. In particular, compound **3b**, identified as the most potent inhibitor against *Pythium aphanidermatum* with an EC_50_ value of 1.6 μg/mL, showed similar activity to commercial Fluopicolide (EC_50_ = 1.0 μg/mL). The further in vivo trial of **3b** is ongoing. SAR analysis shows that heterocycle piperidine plays an important role on fungicidal activity, and the results provide useful clues for further structural modification.

## Figures and Tables

**Table 1:** In vitro fungicidal activities of the title compounds.

Compounds	R^1^	R^2^	Inhibitory Rate (%)/50 μg/mL					
			*P. a*	*R. s*	*V. m*	*B. c*	*A. s*	*G. g*
**3a**	H	2,6-Cl_2_	20	80	86	78	70	61
**3b**	H	2-OH,5-Cl	99	93	86	72	44	99
**3c**	H	4-OCH_2_Ph	31	76	79	50	56	53
**3d**	H	3,5-F_2_	49	87	88	64	70	98
**3e**	H	3-F	55	91	92	60	71	97
**3f**	3-CH_3_	2,6-Cl_2_	52	90	84	69	63	54
**3g**	3-CH_3_	2-OH,5-Cl	65	68	94	58	60	100
**3h**	3-CH_3_	4-OCH_2_Ph	17	74	77	61	45	61
**3i**	3-CH_3_	3,5-F_2_	30	78	83	52	66	81
**3j**	3-CH_3_	3-F	55	95	96	62	73	100
**3k**	3-OH	2,6-Cl_2_	46	89	62	29	38	34
**3l**	3-OH	2-OH,5-Cl	98	79	39	52	43	63
**3m**	3-OH	4-OCH_2_Ph	29	76	57	58	47	28
**3n**	3-OH	3,5-F_2_	36	52	49	17	31	22
**3o**	3-OH	3-F	27	67	47	8	23	11
**3p**	4-COOC_2_H_5_	2,6-Cl_2_	47	80	78	48	52	76
**3q**	4-COOC_2_H_5_	2-OH,5-Cl	48	61	65	49	47	31
**3r**	4-COOC_2_H_5_	4-OCH_2_Ph	15	71	61	52	32	98
**3s**	4-COOC_2_H_5_	3,5-F_2_	17	40	65	7	32	1
**3t**	4-COOC_2_H_5_	3-F	35	82	70	37	54	55
**3u**	4-COOH	2,6-Cl_2_	14	75	40	25	17	15
**3v**	4-COOH	2-OH,5-Cl	11	66	96	75	54	30
**3w**	4-COOH	4-OCH_2_Ph	22	74	67	61	42	42
**3x**	4-COOH	3,5-F_2_	0	12	38	51	23	7
**3y**	4-COOH	3-F	0	51	35	51	43	0
Fluopicolide	-	-	99	43	63	43	58	95

*P. a*: *Pythium aphanidermatum*; *R. s*: *Rhizoctonia solani*; *V. m*: *Valsa mali*; *B. c*: *Botrytis cinerea*; *A. s*: *Alternaria solani*; *G. g*: *Gaeu-mannomyces graminsis*.

**Table 2:** The EC_50_ value of some title compounds.

Compounds	R^1^	R^2^	EC_50_ (μg/mL)			
			*R. s*	*V. m*	*G. g*	*P. a*
**3a**	H	2,6-Cl_2_	15.2	10.5	NT ^a^	NT
**3b**	H	2-OH,5-Cl	9.6	2.3	9.3	1.6
**3c**	H	4-OCH_2_Ph	2.2	4.6	NT	NT
**3d**	H	3,5-F_2_	11.6	12.2	17.2	NT
**3e**	H	3-F	8.4	18.1	14.9	NT
**3f**	3-CH_3_	2,6-Cl_2_	2.1	35.1	NT	NT
**3g**	3-CH_3_	2-OH,5-Cl	NT	2.8	10.1	NT
**3i**	3-CH_3_	3,5-F_2_	8.3	5.3	18.1	NT
**3j**	3-CH_3_	3-F	8.0	9.0	16.5	NT
**3l**	3-OH	2-OH,5-Cl	26.3	NT	NT	4.3
**3q**	4-COOC_2_H_5_	2,6-Cl_2_	9.2	14.3	48.9	NT
**3r**	4-COOC_2_H_5_	4-OCH_2_Ph	NT	NT	72.0	NT
**3t**	4-COOC_2_H_5_	3-F	10.6	NT	NT	NT
**3v**	4-COOH	2-OH,5-Cl	NT	9.9	NT	NT
**3w**	4-COOH	4-OCH_2_Ph	NT	NT	59.5	NT
**3z**		2-OH,5-Cl	>30.0	17.2	24.8	22.8
Azoxystrobin			NT	0.01	NT	16.9
Pyraclostrobin			0.03	0.01	0.19	NT
Fluopicolide			NT	NT	NT	1.0

^a^ NT represent not tested. *R. s*: *Rhizoctonia solani*; *V. m*: *Valsa mali*; *G. g*: *Gaeu-mannomyces graminsis*; *P. a*: *Pythium aphanidermatum*.

## Supplementary Materials

The following are available online. Figures S1–S53: ^1^H-NMR spectra of compounds **1a**, **2a**, **3a**–**3y**, ^13^C-NMR spectra of compounds **3a**–**3y**, DEPT-135 spectra of compound **3b**.
