# Supplementary material for: Design, Synthesis, and Fungicidal Activity of Novel Thiosemicarbazide Derivatives Containing Piperidine Fragments
Source: Molecules. 2017 Dec 11;22(12):2085. doi: 10.3390/molecules22122085 (PMC6149837; doi:10.3390/molecules22122085)
Supplement: Supplementary file 1 [file molecules-22-02085-s001.pdf]

Supporting information

# Design, Synthesis, and Fungicidal Activity of Novel Thiosemicarbazide Derivatives Containing Piperidine Fragments

**Xuebo Zhang, Peng Lei, Tengda Sun, Xiaoyu Jin, Xinling Yang and Yun Ling ***

Department of Applied Chemistry, College of Science, China Agricultural University, Beijing 100193, China; zhangxueb@cau.edu.cn (X.Z.); leipeng@cau.edu.cn (P.L.); tenda@cau.edu.cn (T.S.); stellajxy@cau.edu.cn (X.J.); yangxl@cau.edu.cn (X.Y.)

* Correspondence: lyun@cau.edu.cn; Tel.: +86-(0)10-6273-2223

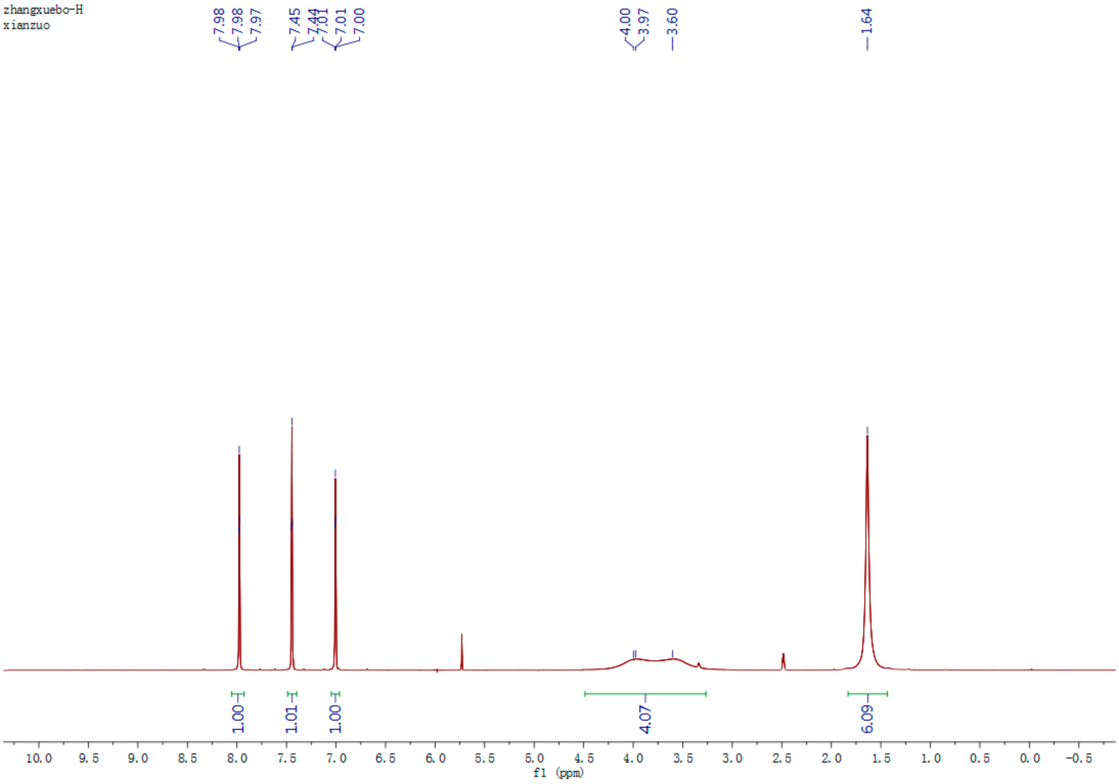


**Figure S1.** $^{1}$H-NMR spectra of the intermediate compound (1H-imidazol-1-yl)(piperidin-1-yl)methanethione (**1a**).

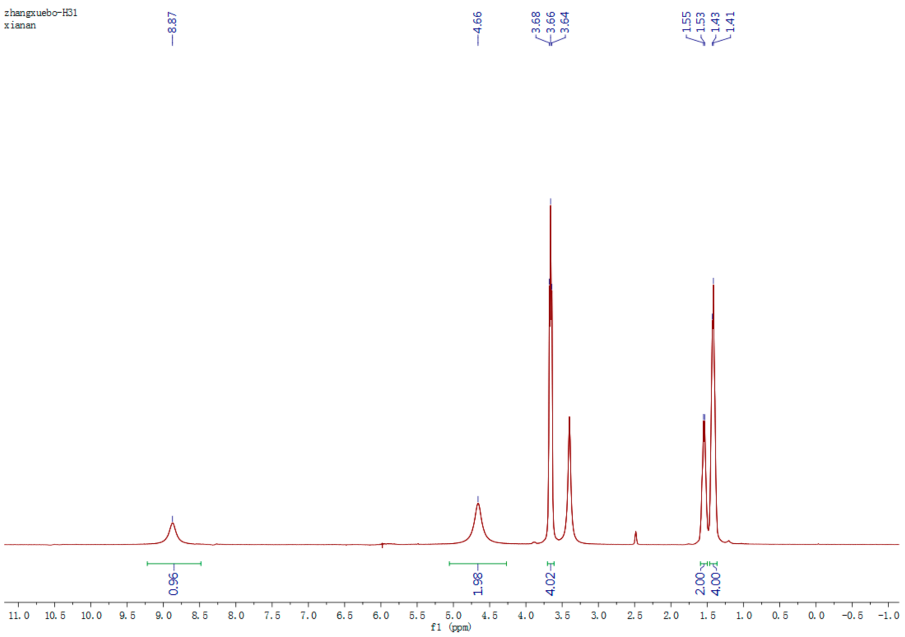


**Figure S2.** $^1$H-NMR spectra of the intermidate compound piperidine-1-carbothiohydrazide (**2a**).

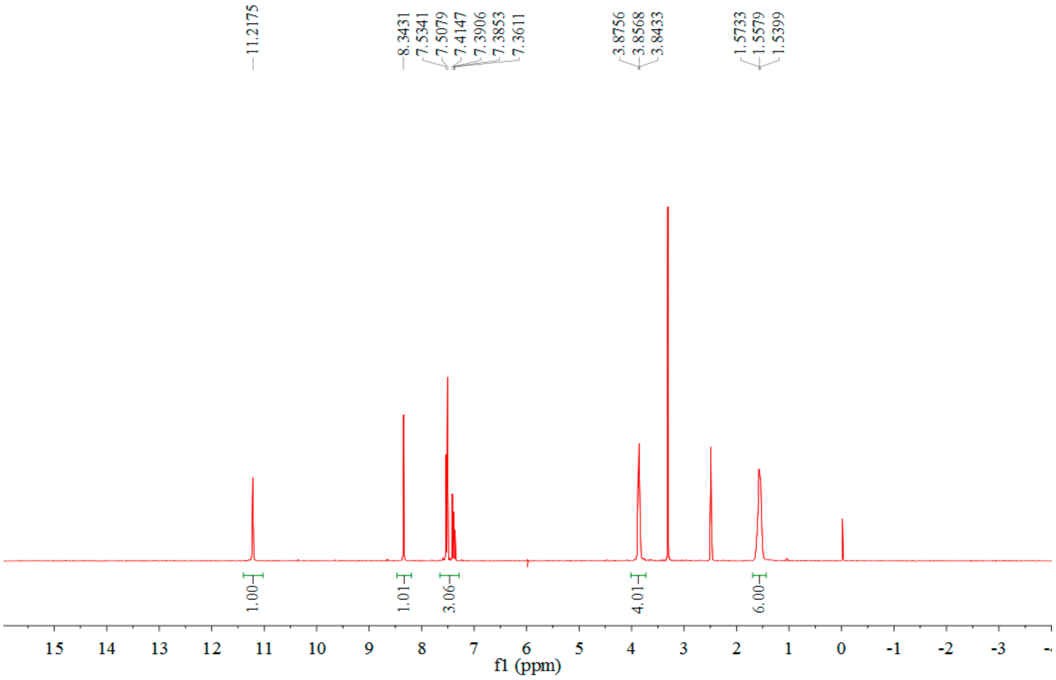


**Figure S3.** $^1$H-NMR spectra of the title compound (*E*)-*N*'-(2,6-dichlorobenzylidene)piperidine-1-carbothiohydrazide (**3a**).

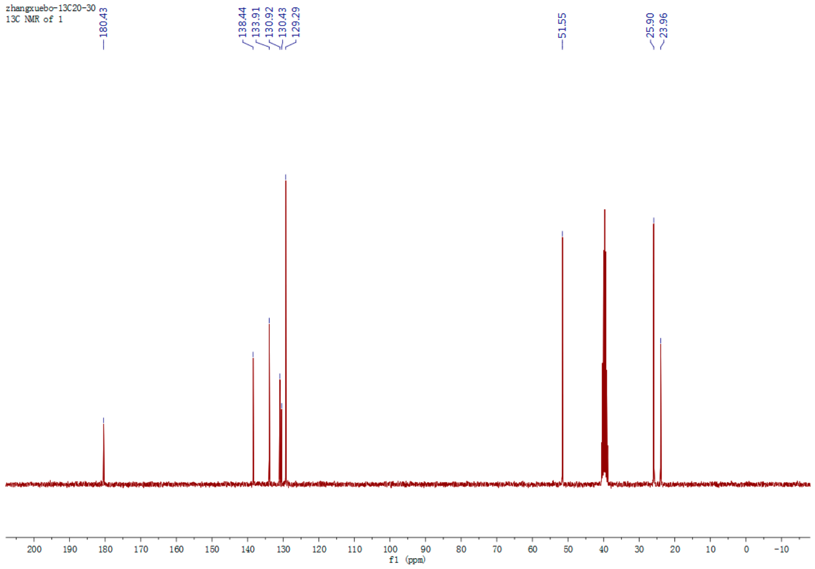

**Figure S4.** $^{13}$C-NMR spectra of the title compound (*E*)-*N*'-(2,6-dichlorobenzylidene)piperidine-1- carbothiohydrazide (**3a**).

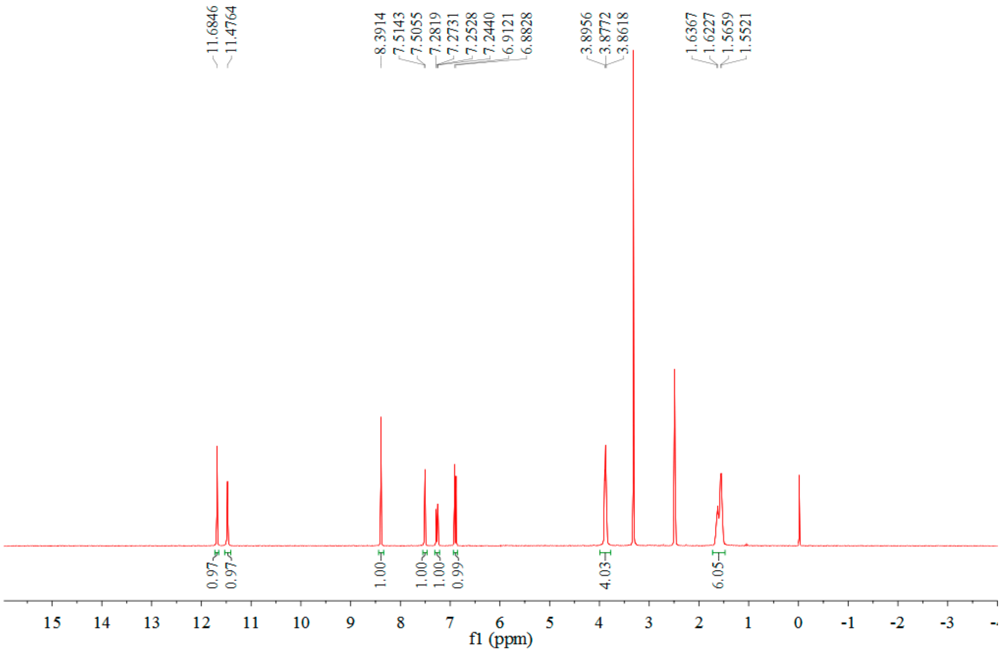

**Figure S5.** $^{1}$H NMR spectra of the title compound (*E*)-*N*'-(5-chloro-2-hydroxybenzylidene)piperidine-1- carbothiohydrazide (**3b**)

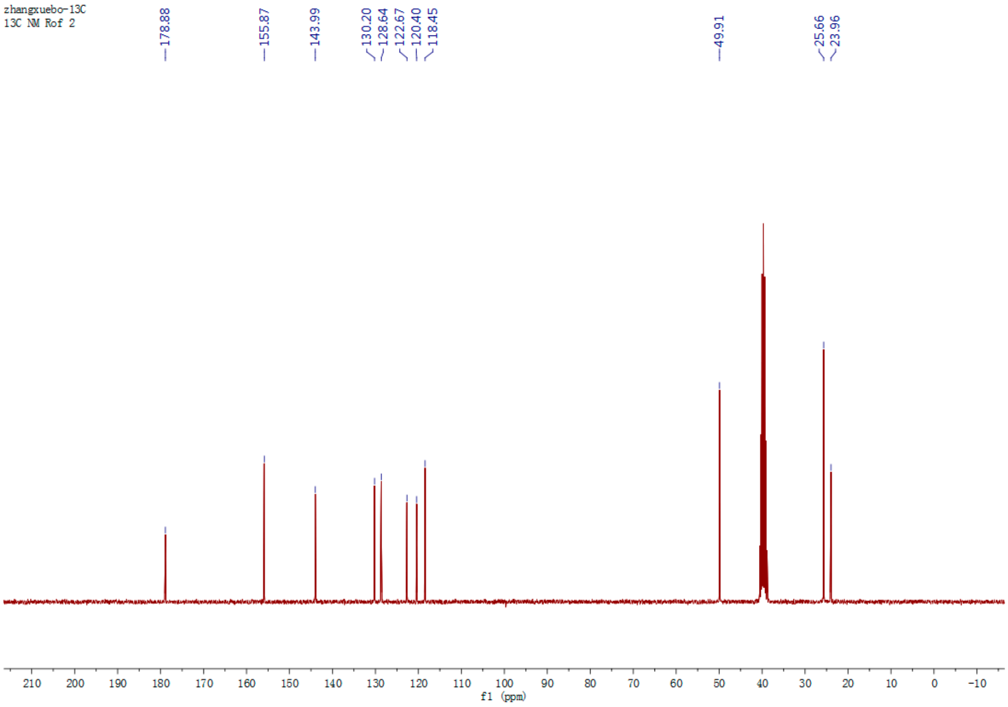


**Figure S6.** $^{13}C$ NMR spectra of the title compound **(*E*)-*N*'-(5-chloro-2-hydroxybenzylidene)piperidine-1- carbothiohydrazide (3b)**

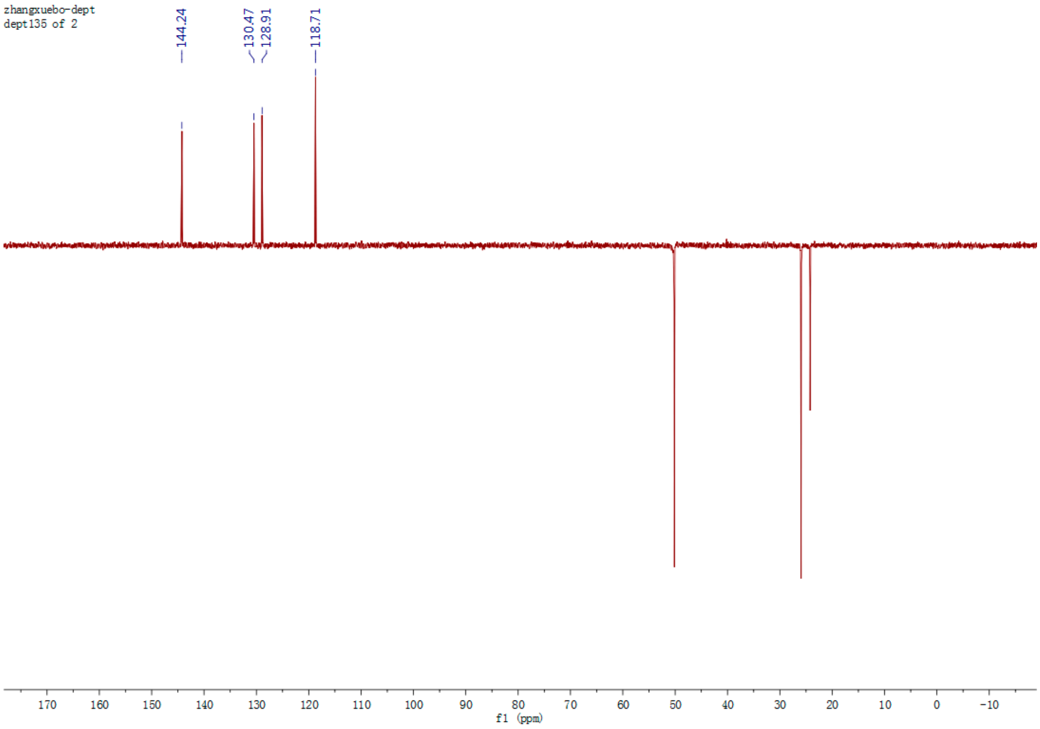


**Figure S7.** DEPT 135spectra of the title compound **(*E*)-*N*'-(5-chloro-2-hydroxybenzylidene)piperidine-1- carbothiohydrazide (3b)**

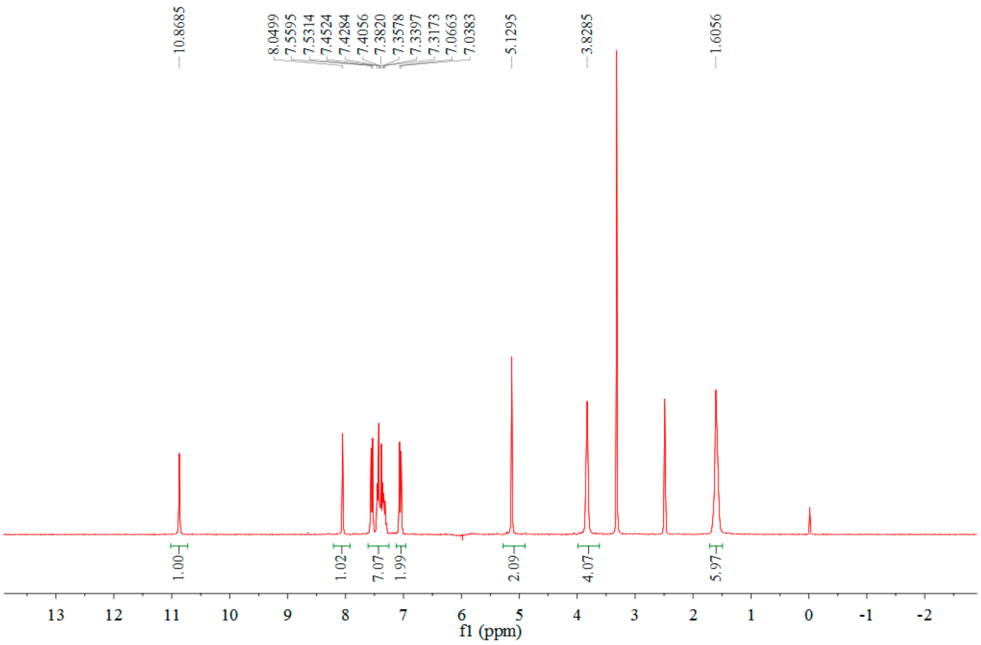

**Figure S8.** $^{1}H$ NMR spectra of the title compound **(*E*)-*N*'-(4-(benzyloxy)benzylidene)piperidine-1-carbothiohydrazide (3c)**

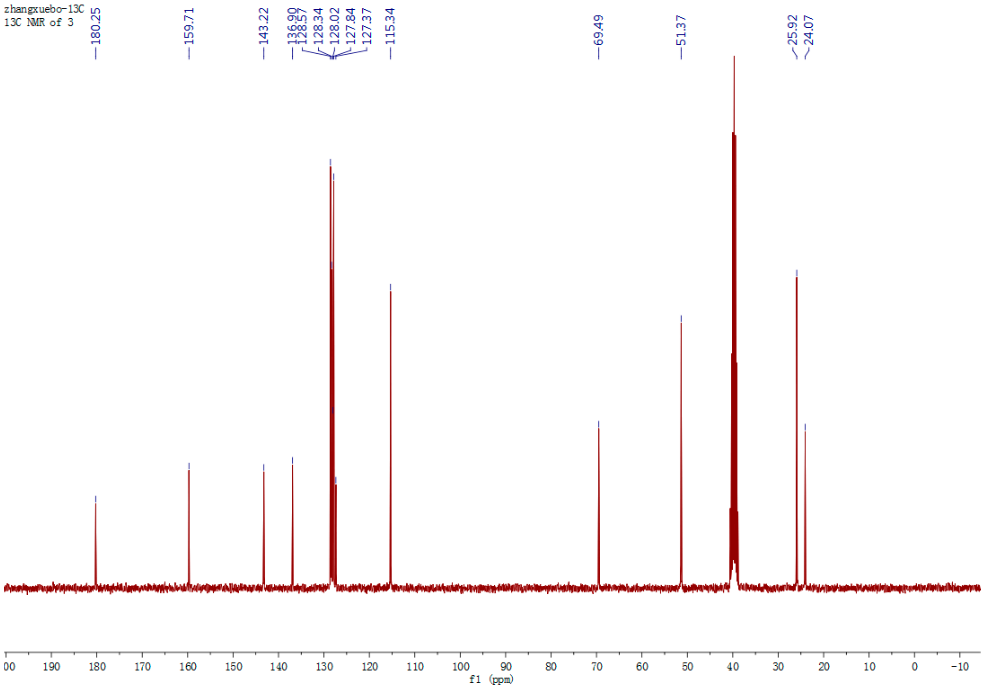

**Figure S9.** $^{13}C$ NMR spectra of the title compound**(*E*)-*N*'-(4-(benzyloxy)benzylidene)piperidine-1-carbothiohydrazide (3c)**

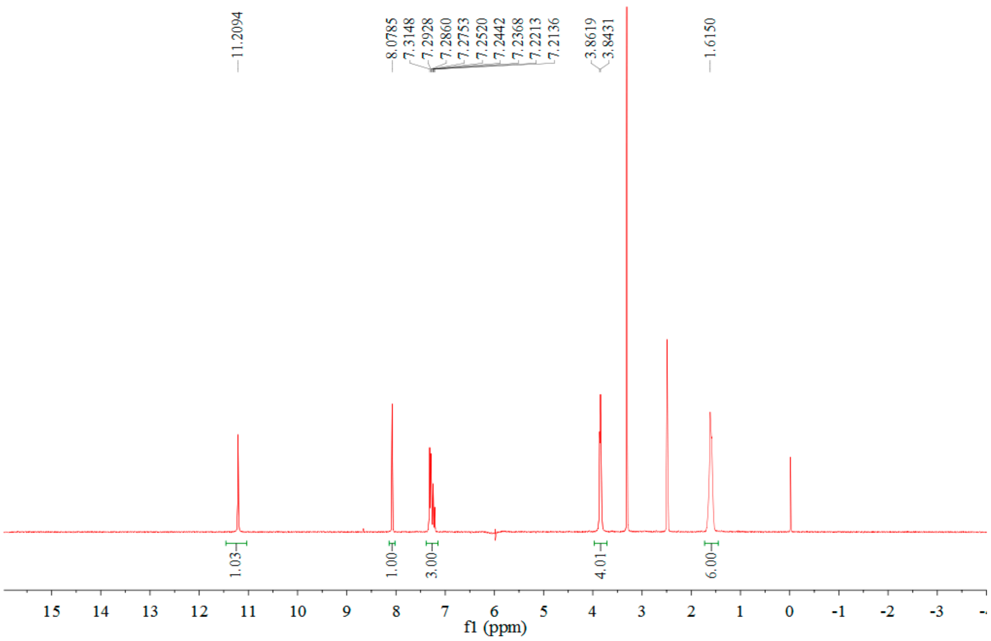


**Figure S10.** $^{1}$H NMR spectra of the title compound **(*E*)-*N'*-(3,5-difluorobenzylidene)piperidine-1-carbothiohydrazide(3d)**

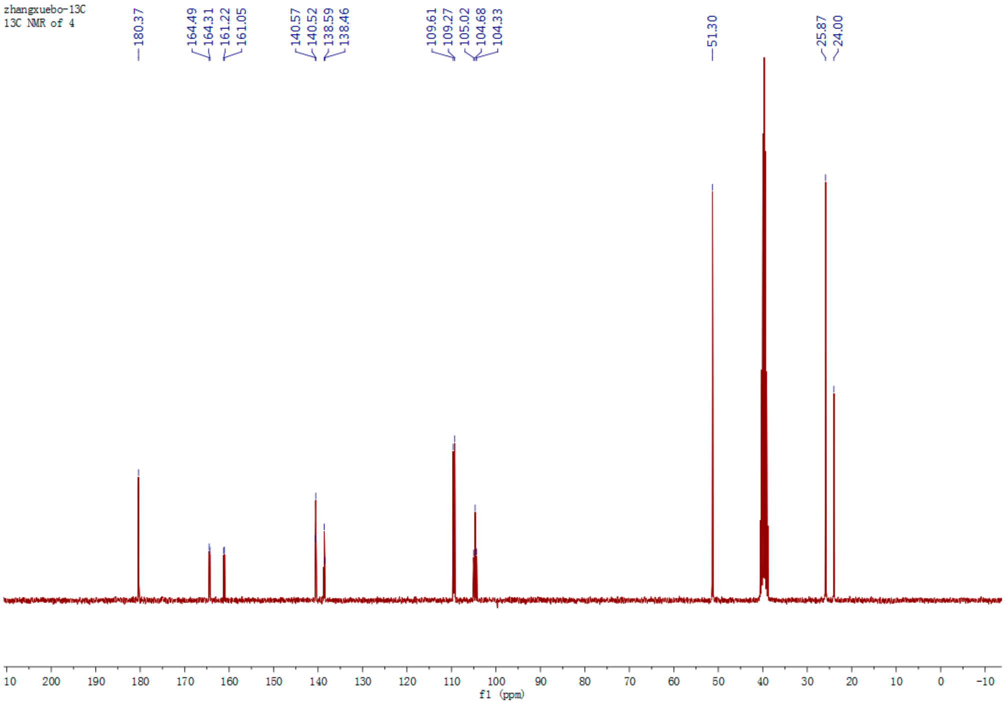


**Figure S11.** $^{13}$C NMR spectra of the title compound**(*E*)-*N'*-(3,5-difluorobenzylidene)piperidine-1-carbothiohydrazide(3d).**

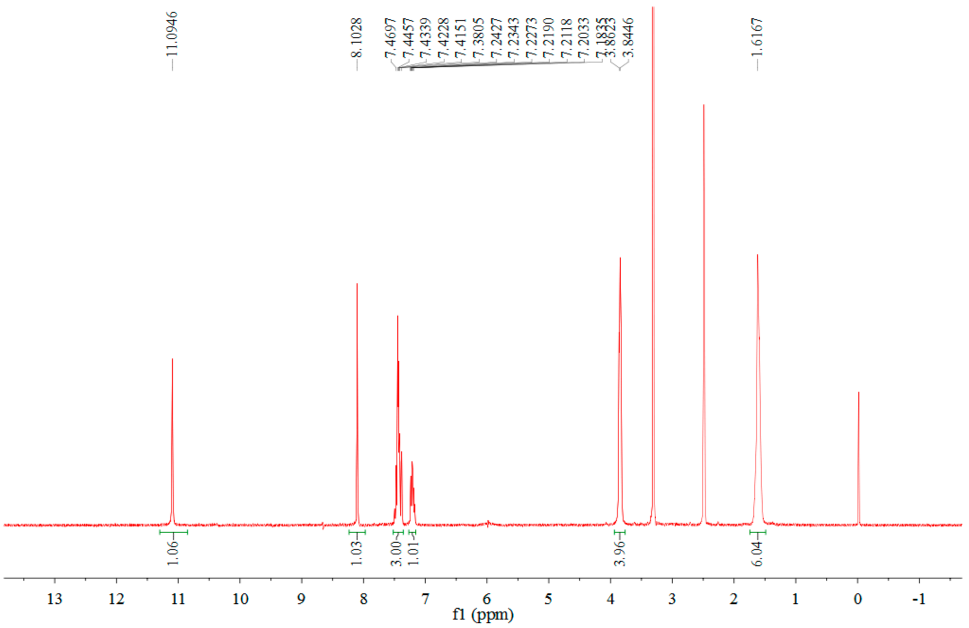

**Figure S12.** ¹H NMR spectra of the title compound **(*E*)-*N*'-(3-fluorobenzylidene)piperidine-1-carbothiohydrazide (3e)**

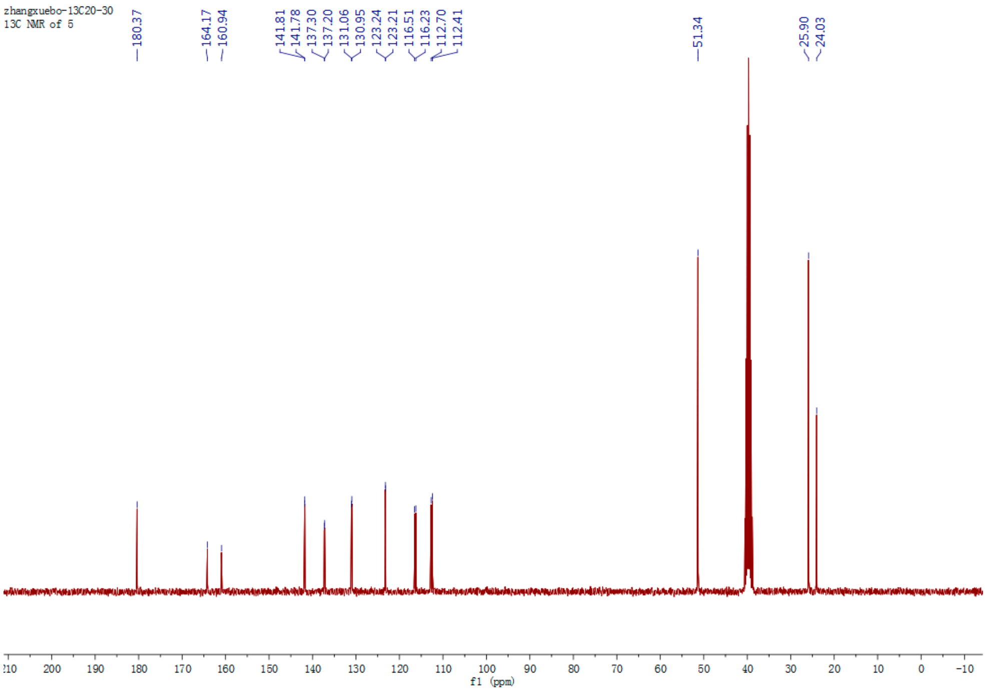

**Figure S13.** ¹³C NMR spectra of the title compound**(*E*)-*N*'-(3-fluorobenzylidene)piperidine-1-carbothiohydrazide (3e)**

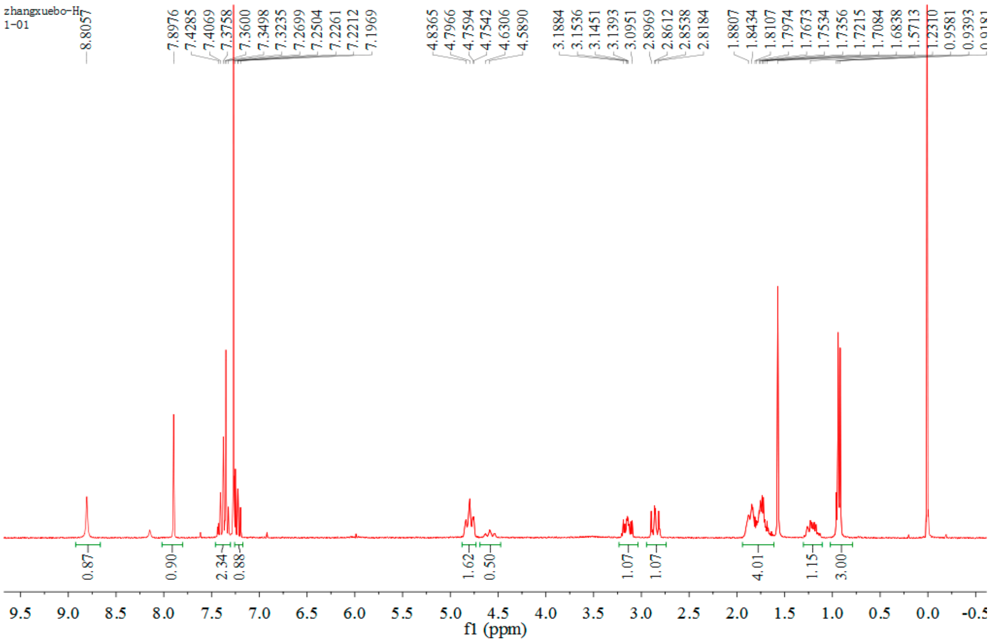


**Figure S14.** $^{1}H$ NMR spectra of the title compound **(*E*)-*N*'-(2,6-dichlorobenzylidene)-3-methylpiperidine-1-carbothiohydrazide (3f)**

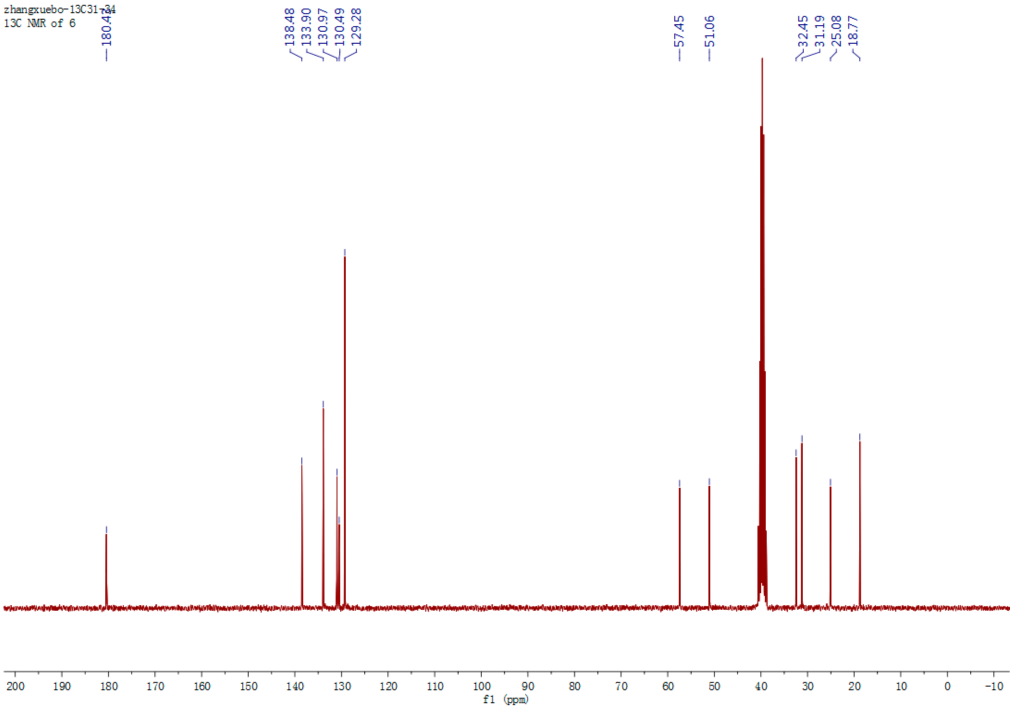


**Figure S15.** $^{13}C$ NMR spectra of the title compound**(*E*)-*N*'-(2,6-dichlorobenzylidene)-3-methylpiperidine-1-carbothiohydrazide (3f)**

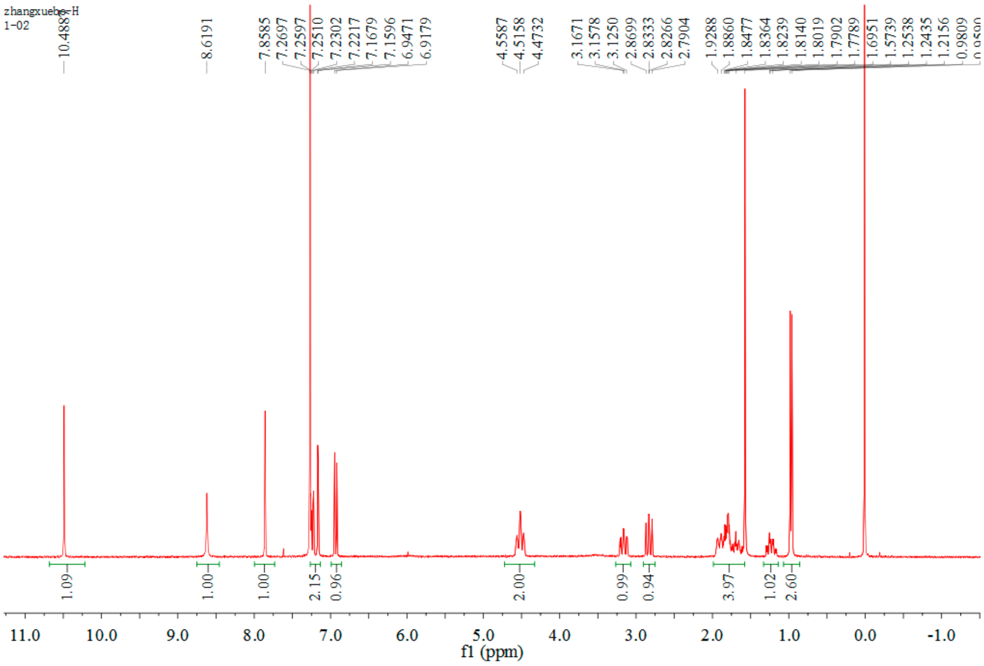


**Figure S16.** ¹H NMR spectra of the title compound(***E*)-*N*'-(5-chloro-2-hydroxybenzylidene)-3-methylpiperidine-1-carbothiohydrazide (3g)**

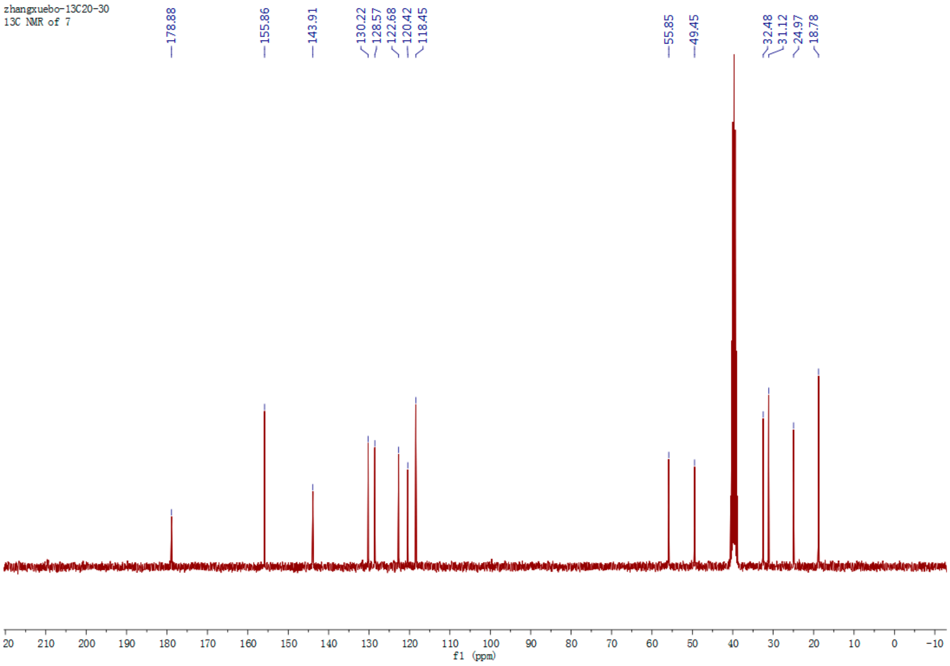


**Figure S17.** ¹³C NMR spectra of the title compound(***E*)-*N*'-(5-chloro-2-hydroxybenzylidene)-3-methylpiperidine-1-carbothiohydrazide (3g)**

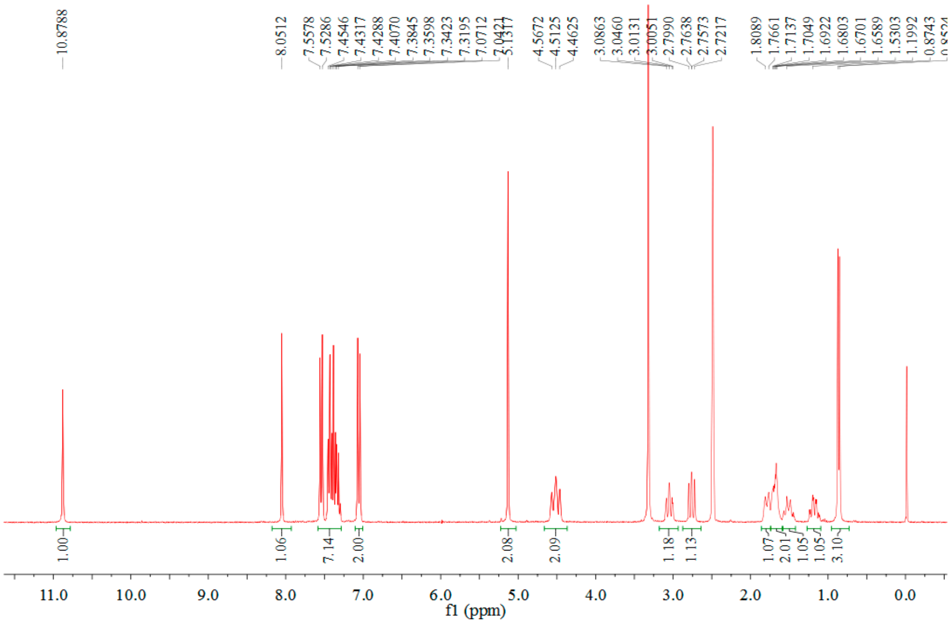


**Figure S18.** $^{1}$H NMR spectra of the title compound**(*E*)-*N*'-(4-(benzyloxy)benzylidene)-3-methylpiperidine-1-carbothiohydrazide**(**3h**)

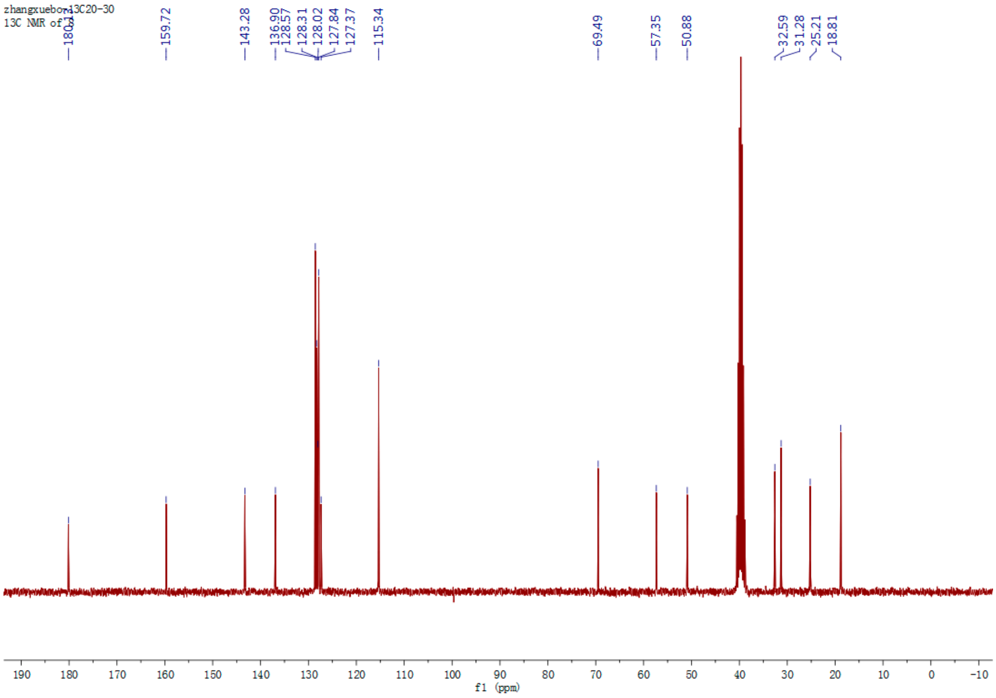


**Figure S19.** $^{13}$C NMR spectra of the title compound**(*E*)-*N*'-(4-(benzyloxy)benzylidene)-3-methylpiperidine-1-carbothiohydrazide**(**3h**)

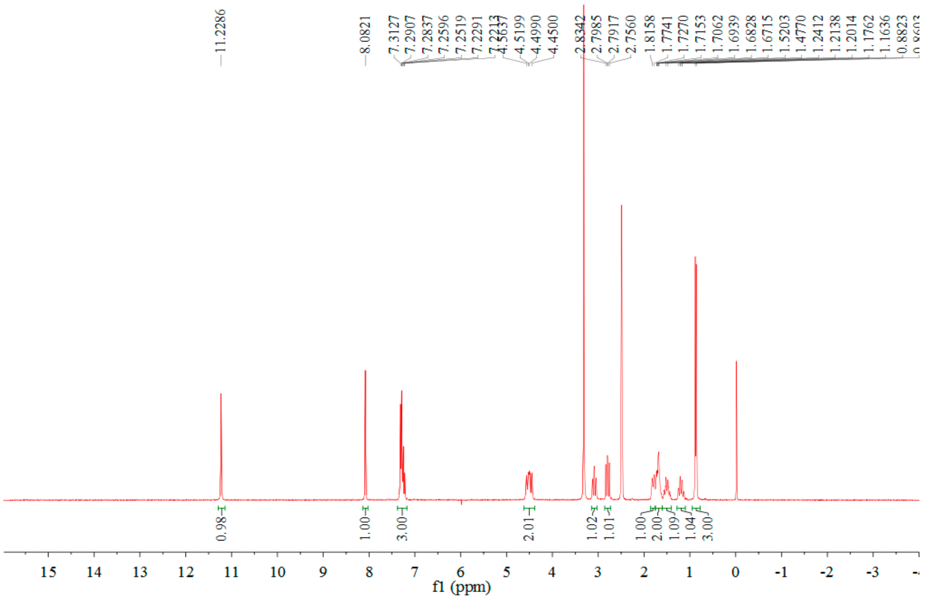


**Figure S20.** $^{1}$H NMR spectra of the title compound**(*E*)-*N*'-(3,5-difluorobenzylidene)-3-methylpiperidine-1-carbothiohydrazide (3i)**

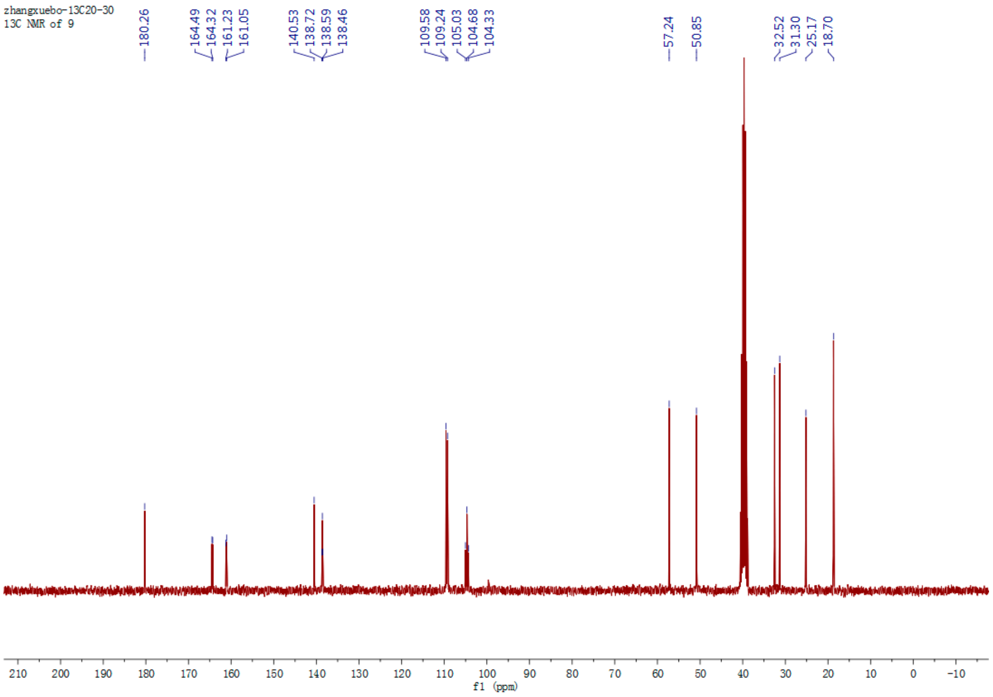


**Figure S21.** $^{13}$C NMR spectra of the title compound**(*E*)-*N*'-(3,5-difluorobenzylidene)-3-methylpiperidine-1-carbothiohydrazide (3i)**

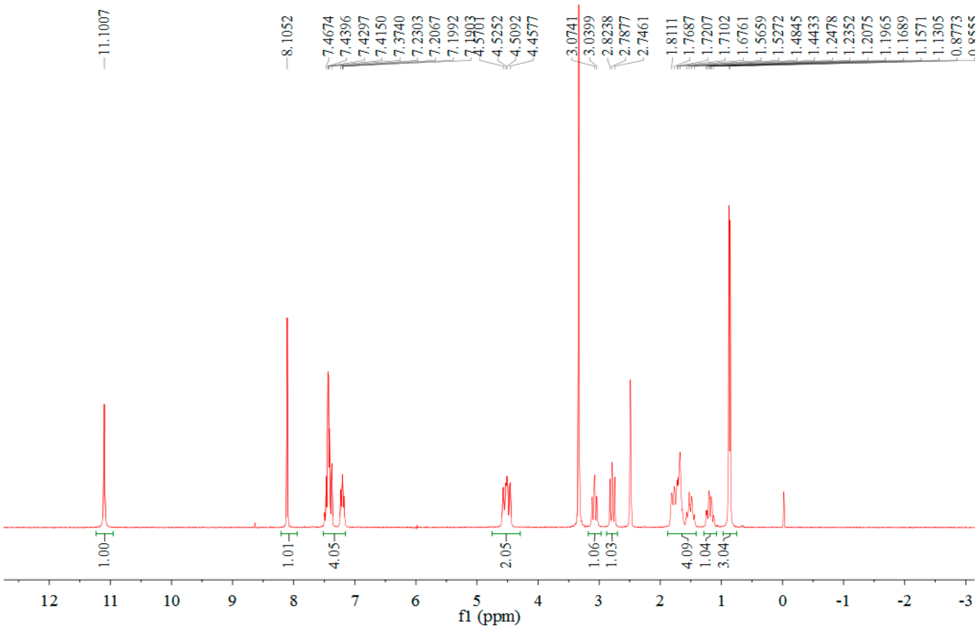


**Figure S22.** $^{1}H$ NMR spectra of the title compound**(*E*)-*N*'-(3-fluorobenzylidene)-3-methylpiperidine-1-carbothiohydrazide (3j)**

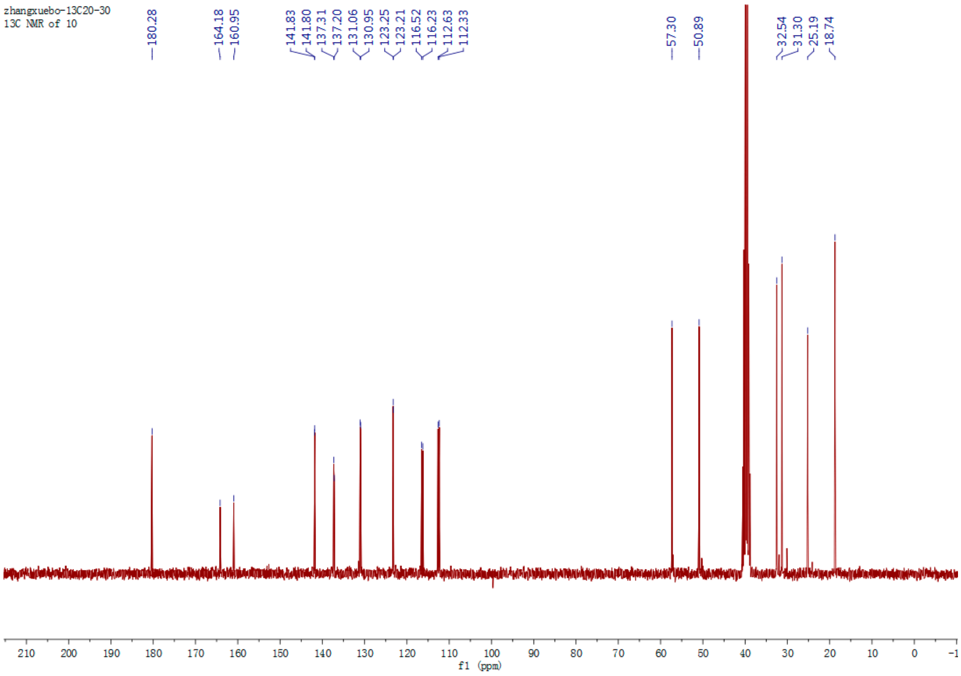


**Figure S23.** $^{13}C$ NMR spectra of the title compound**(*E*)-*N*'-(3-fluorobenzylidene)-3-methylpiperidine-1-carbothiohydrazide (3j)**

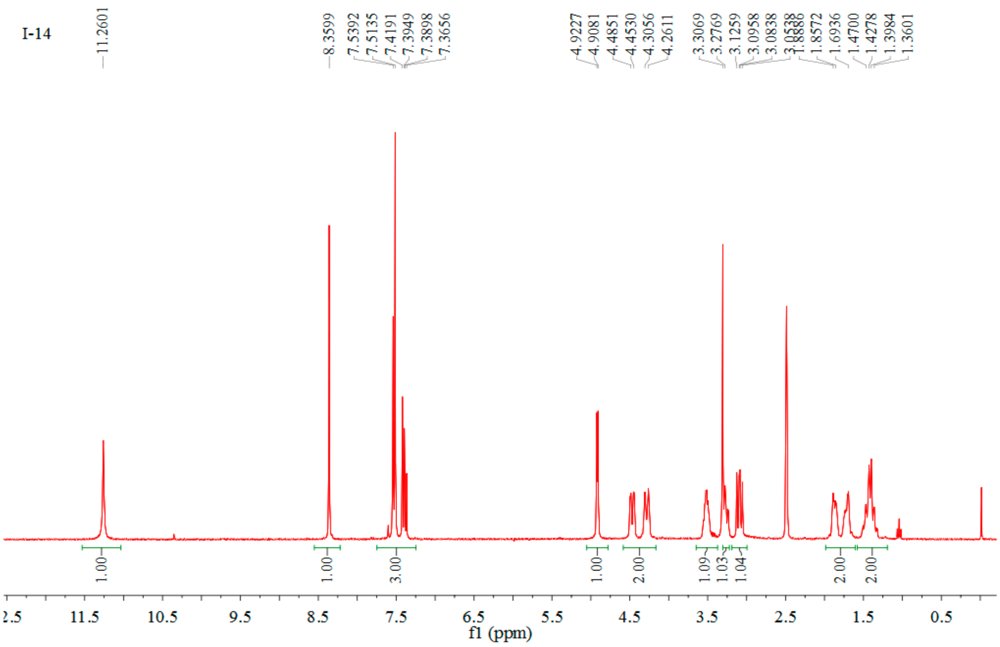


**Figure S24.** $^{1}$H NMR spectra of the title compound**(*E*)-*N*'-(2,6-dichlorobenzylidene)-3-hydroxypiperidine-1-carbothiohydrazide(3k)**

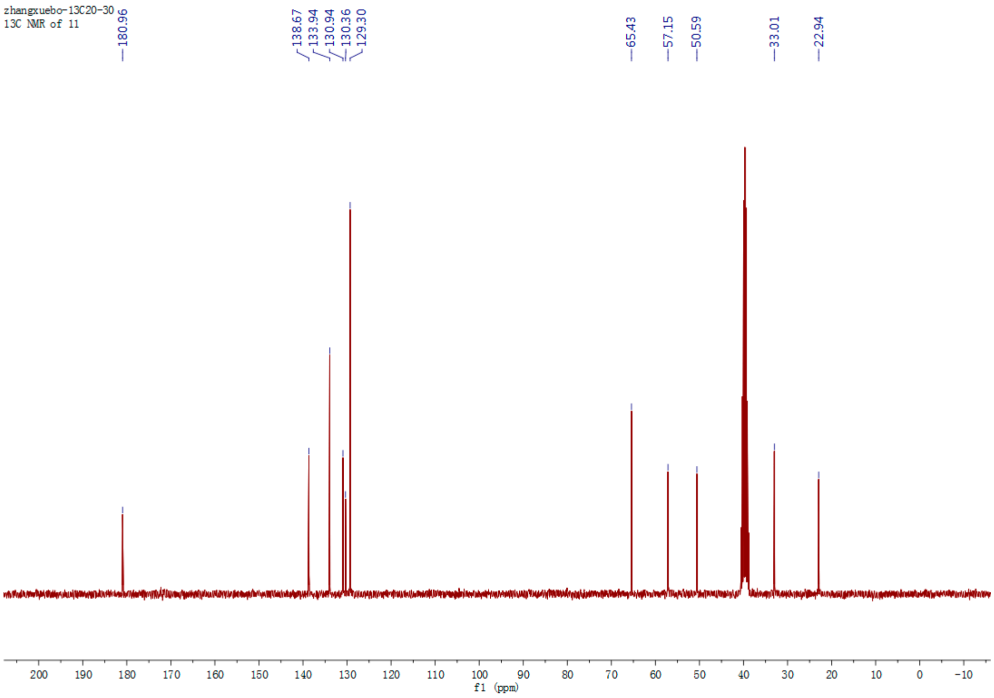


**Figure S25.** $^{13}$C NMR spectra of the title compound**(*E*)-*N*'-(2,6-dichlorobenzylidene)-3-hydroxypiperidine-1-carbothiohydrazide(3k)**

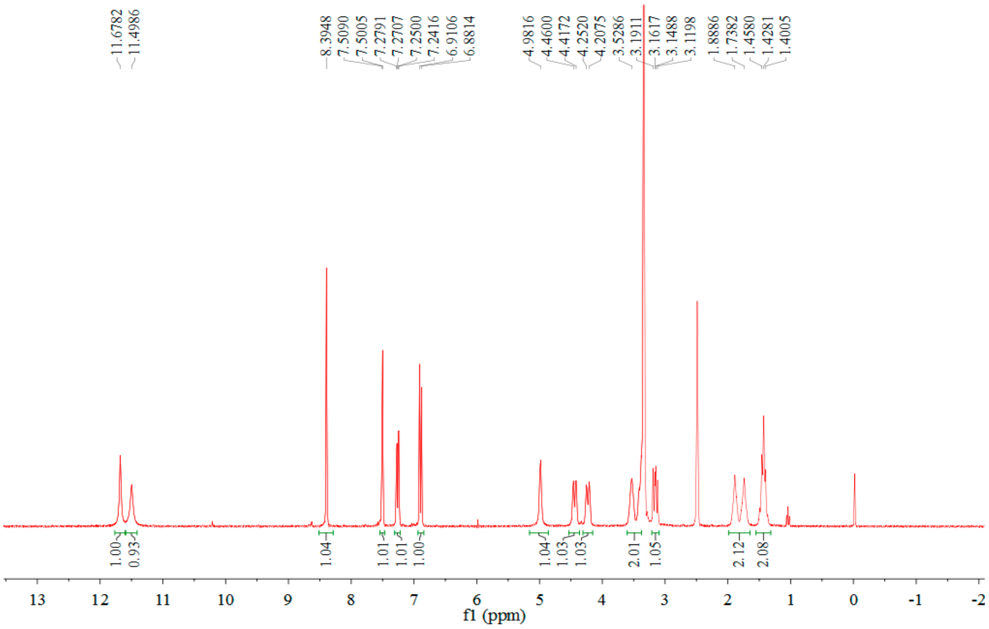


**Figure S26.** $^{1}$H NMR spectra of the title compound**(*E*)-*N*'-(5-chloro-2-hydroxybenzylidene)-3-hydroxypiperidine-1-carbothiohydrazide (3l)**

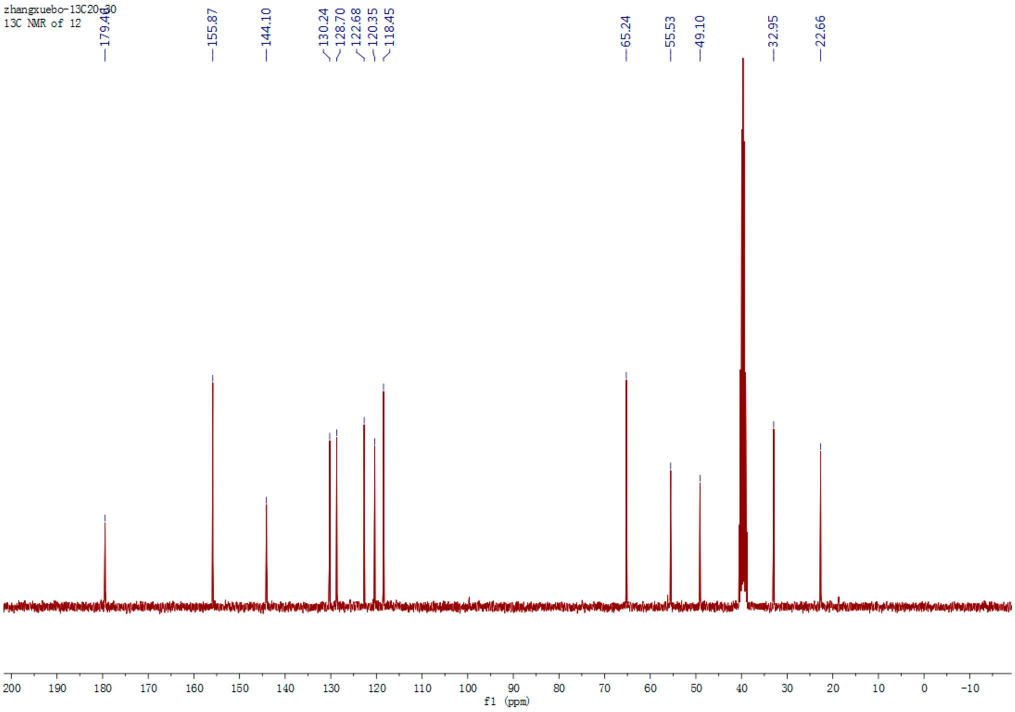


**Figure S27.** $^{13}$C NMR spectra of the title compound**(*E*)-*N*'-(5-chloro-2-hydroxybenzylidene)-3-hydroxypiperidine-1-carbothiohydrazide (3l)**

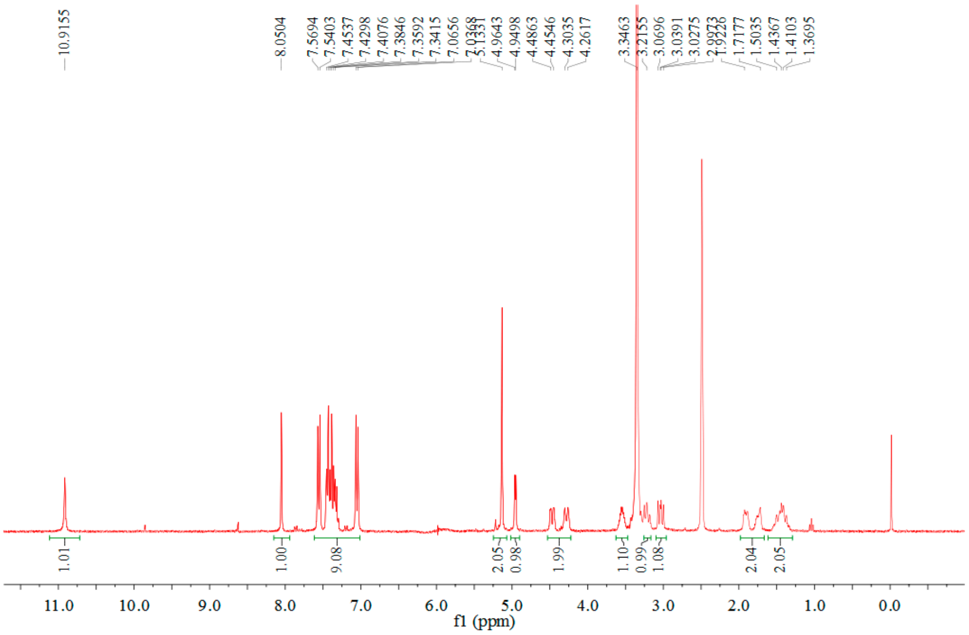

**Figure S28.** ¹H NMR spectra of the title compound**(*E*)-*N*'-(4-(benzyloxy)benzylidene)-3-hydroxypiperidine-1-carbothiohydrazide (3m)**

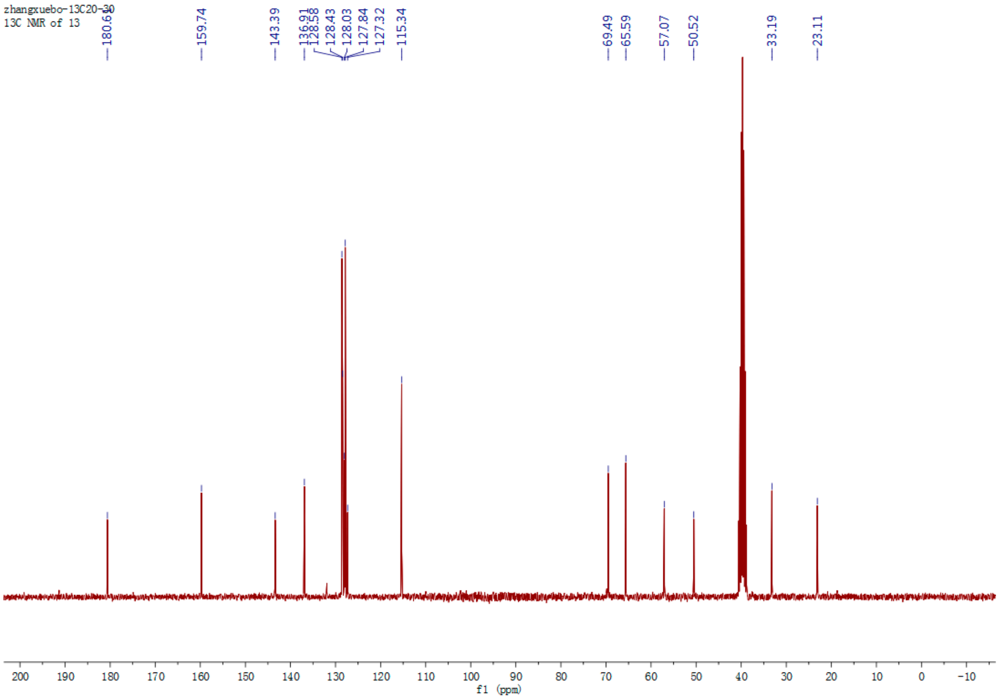


**Figure S29.** ¹³C NMR spectra of the title compound**(*E*)-*N*'-(4-(benzyloxy)benzylidene)-3-hydroxypiperidine-1-carbothiohydrazide (3m)**

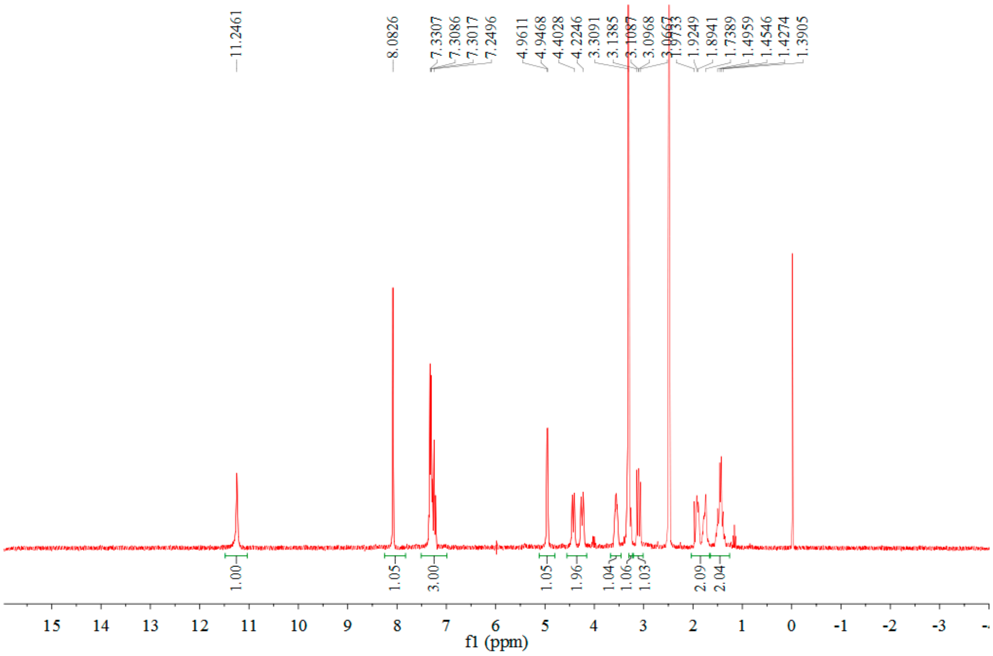


**Figure S30.** $^{1}$H NMR spectra of the title compound**(*E*)-*N*'-(3,5-difluorobenzylidene)-3-hydroxypiperidine-1-carbothiohydrazide (3n)**

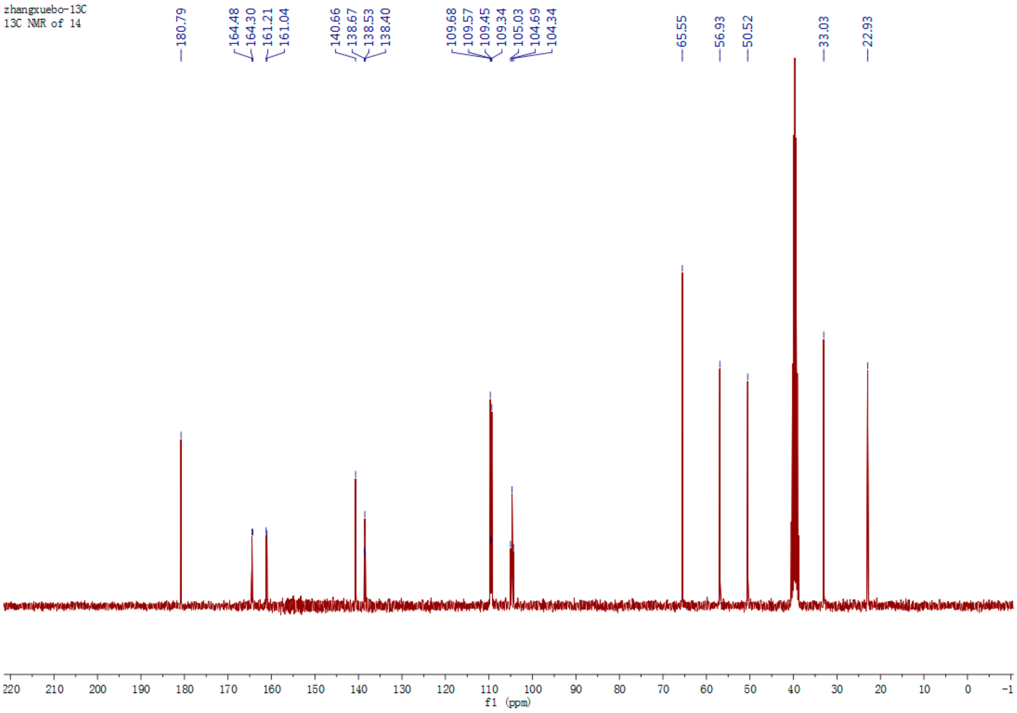


**Figure S31.** $^{13}$C NMR spectra of the title compound**(*E*)-*N*'-(3,5-difluorobenzylidene)-3-hydroxypiperidine-1-carbothiohydrazide (3n)**

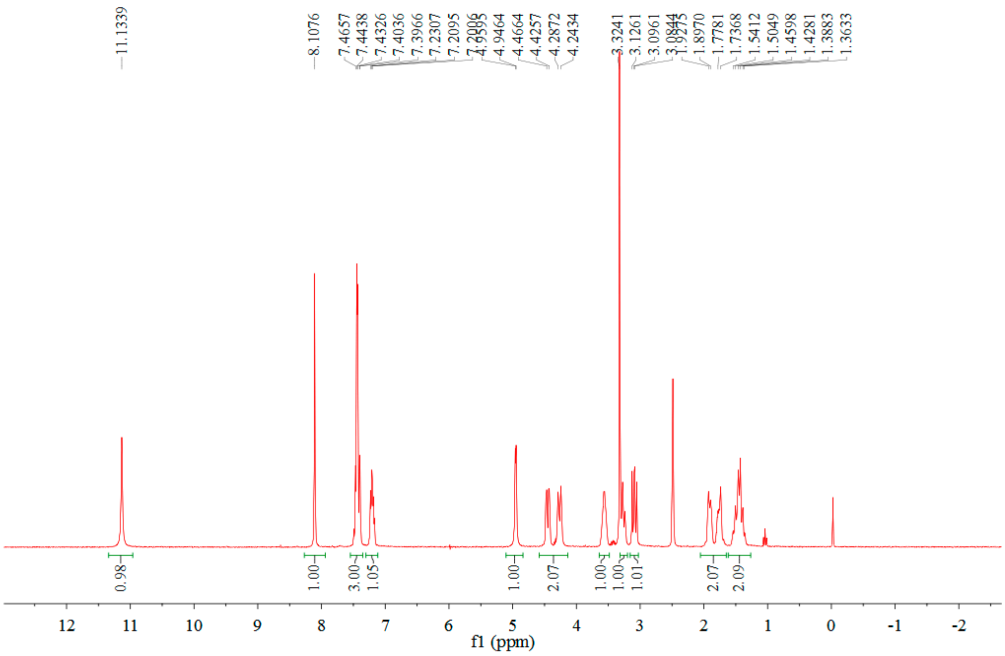


**Figure S32.** $^{1}$H NMR spectra of the title compound**(*E*)-*N*'-(3-fluorobenzylidene)-3-hydroxypiperidine-1-carbothiohydrazide (3o)**

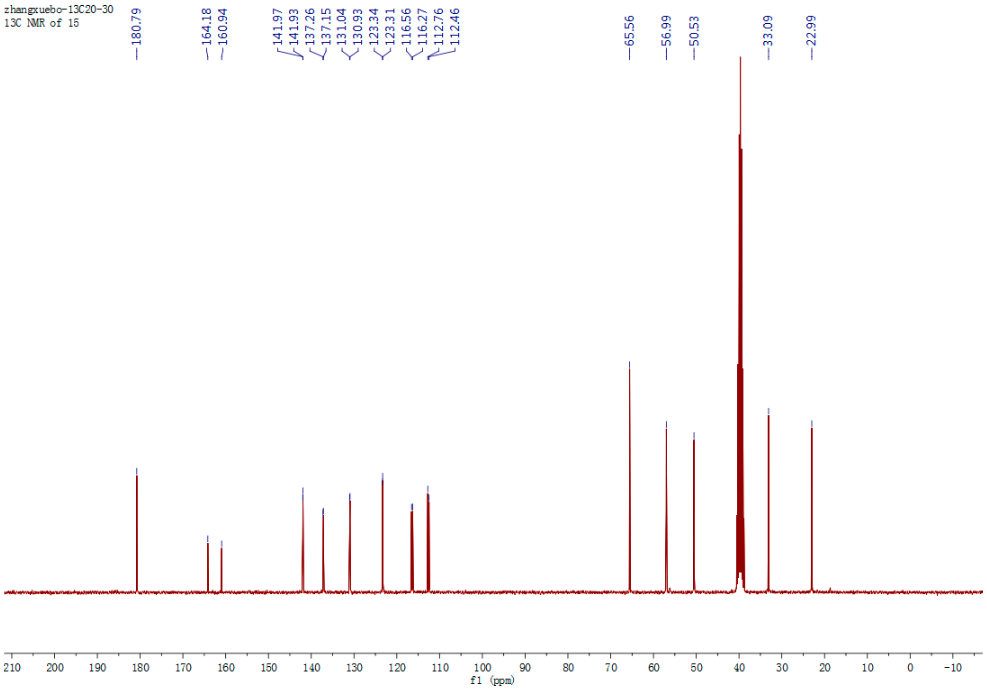


**Figure S33.** $^{13}$C NMR spectra of the title compound**(*E*)-*N*'-(3-fluorobenzylidene)-3-hydroxypiperidine-1-carbothiohydrazide (3o)**

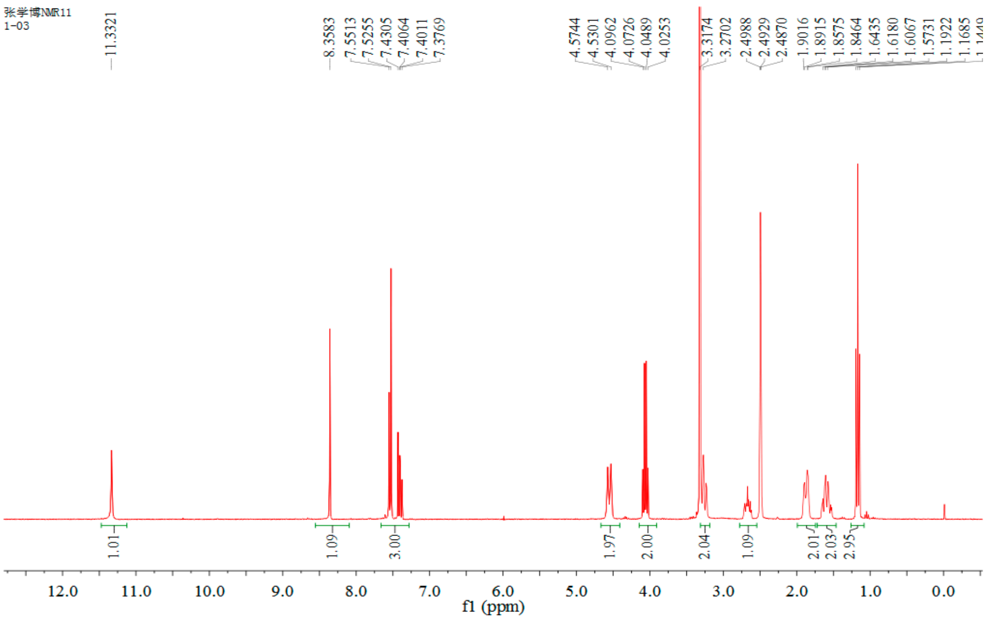


**Figure S34.** ¹H NMR spectra of the title compound **Ethyl (*E*)-1-(2-(2,6-dichlorobenzylidene) hydrazine-1-carbonothioyl)piperidine-4-carboxylate (3p)**

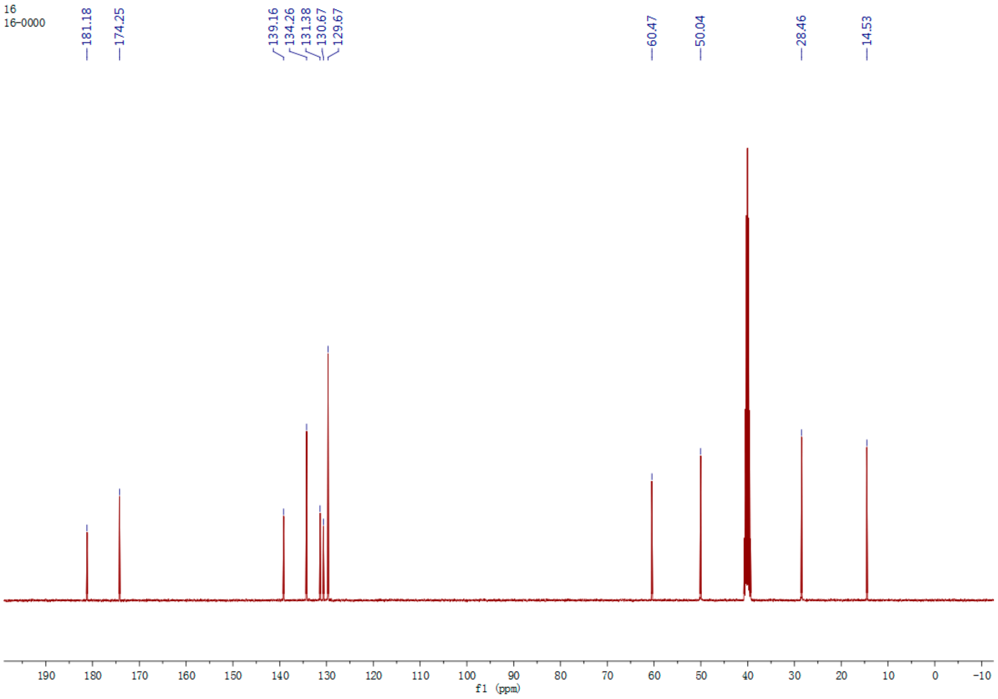


**Figure S35.** ¹³C NMR spectra of the title compound **Ethyl (*E*)-1-(2-(2,6-dichlorobenzylidene) hydrazine-1-carbonothioyl)piperidine-4-carboxylate (3p)**

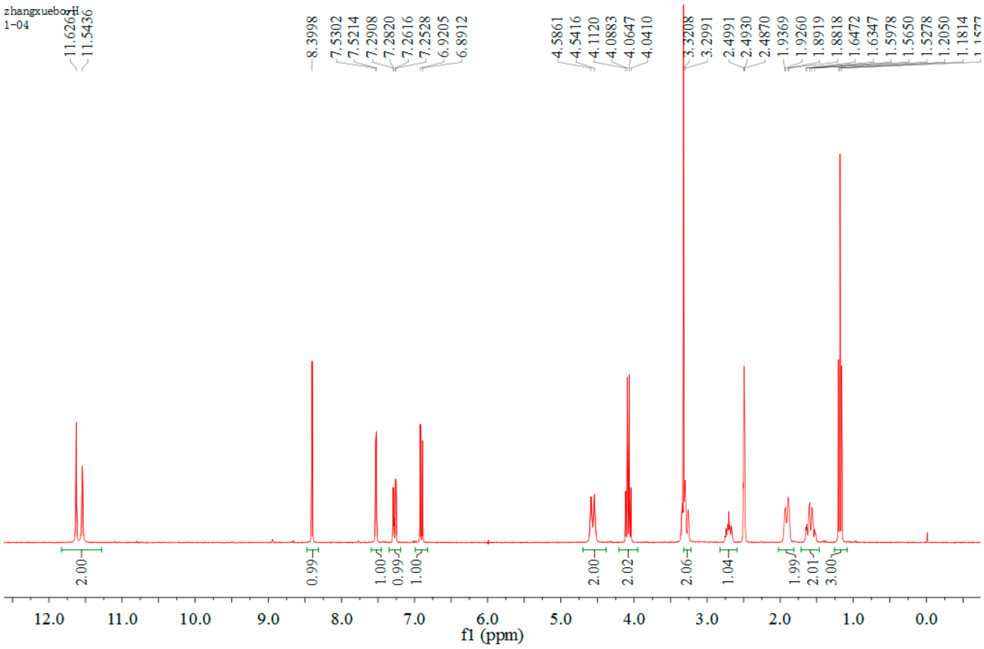


**Figure S36.** $^1H$ NMR spectra of the title compound **Ethyl (*E*)-1-(2-(5-chloro-2-hydroxybenzylidene) hydrazine-1-carbonothioyl)piperidine-4-carboxylate (3q)**

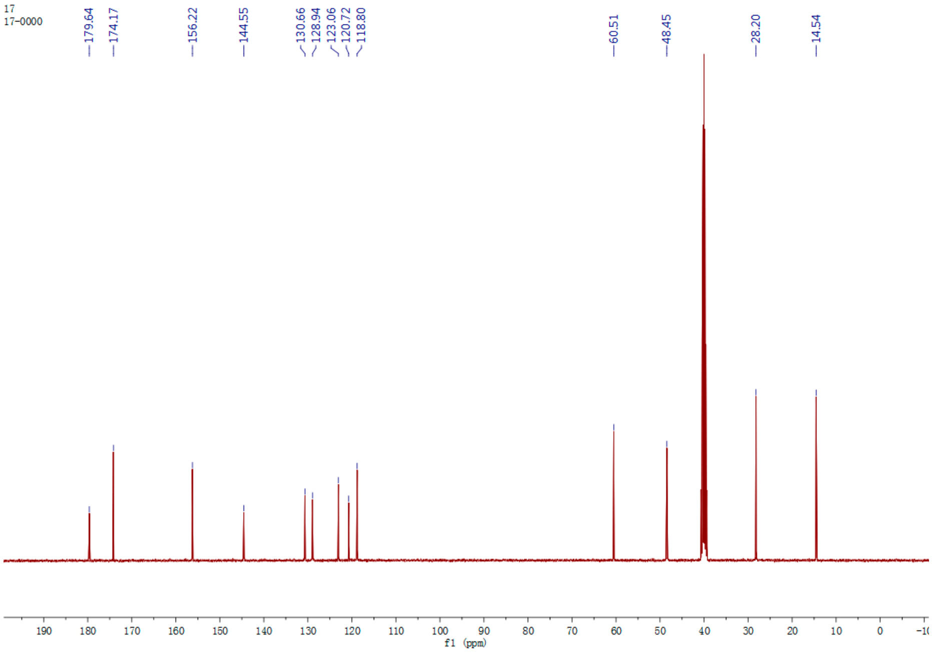


**Figure S37.** $^{13}C$ NMR spectra of the title compound **Ethyl (*E*)-1-(2-(5-chloro-2-hydroxybenzylidene) hydrazine-1-carbonothioyl)piperidine-4-carboxylate (3q)**

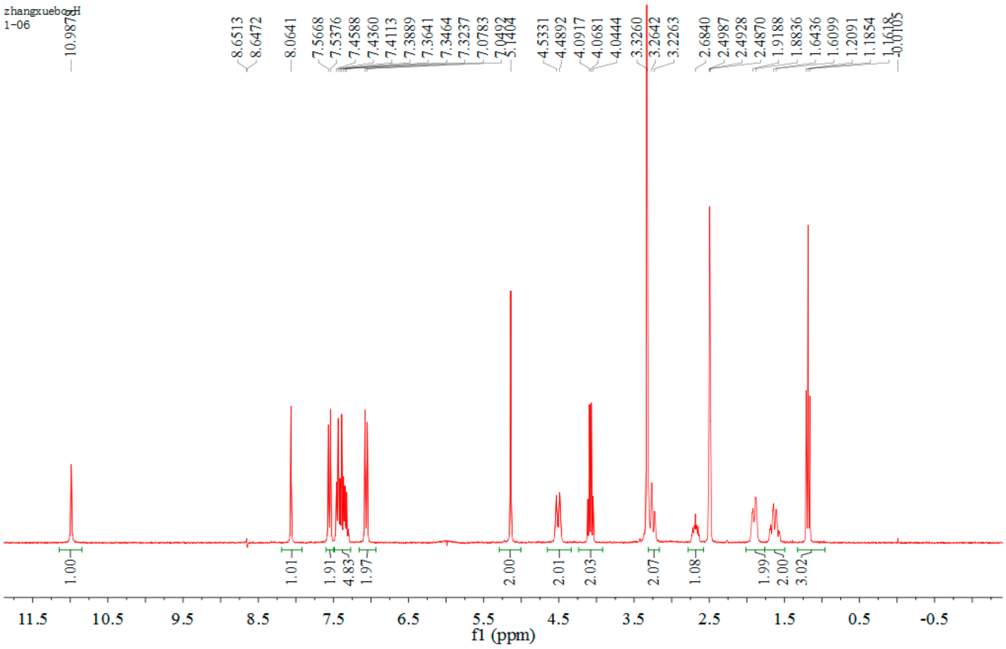

**Figure S38.** $^{1}H$ NMR spectra of the title compound **Ethyl (*E*)-1-(2-(4-(benzyloxy)benzylidene) hydrazine-1-carbonothioyl)piperidine-4-carboxylate (3r)**

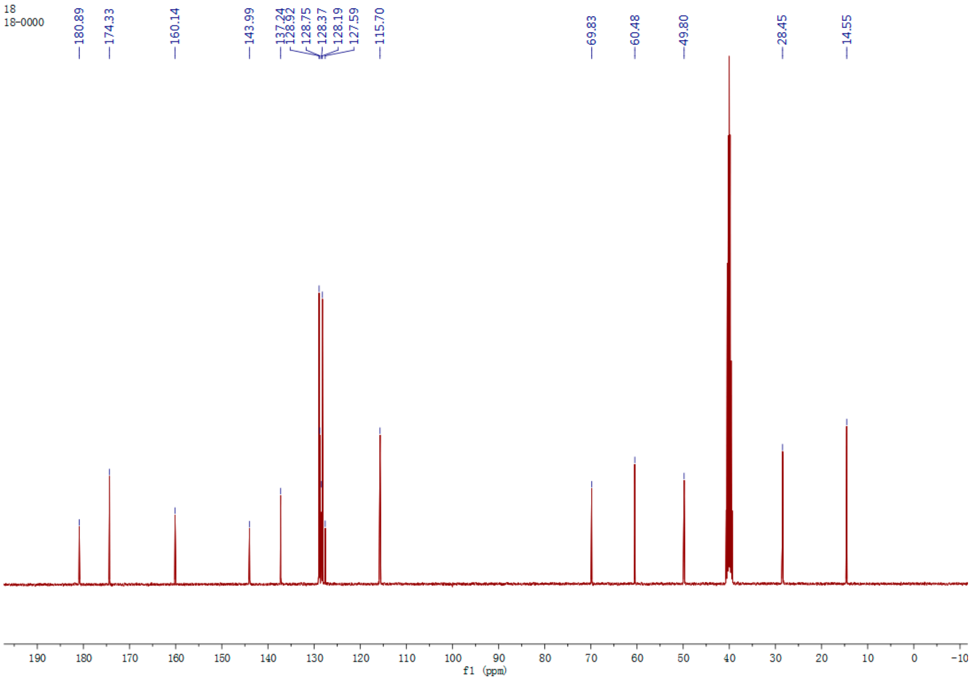

**Figure S39.** $^{13}C$ NMR spectra of the title compound **Ethyl (*E*)-1-(2-(4-(benzyloxy)benzylidene) hydrazine-1-carbonothioyl)piperidine-4-carboxylate** (3r)

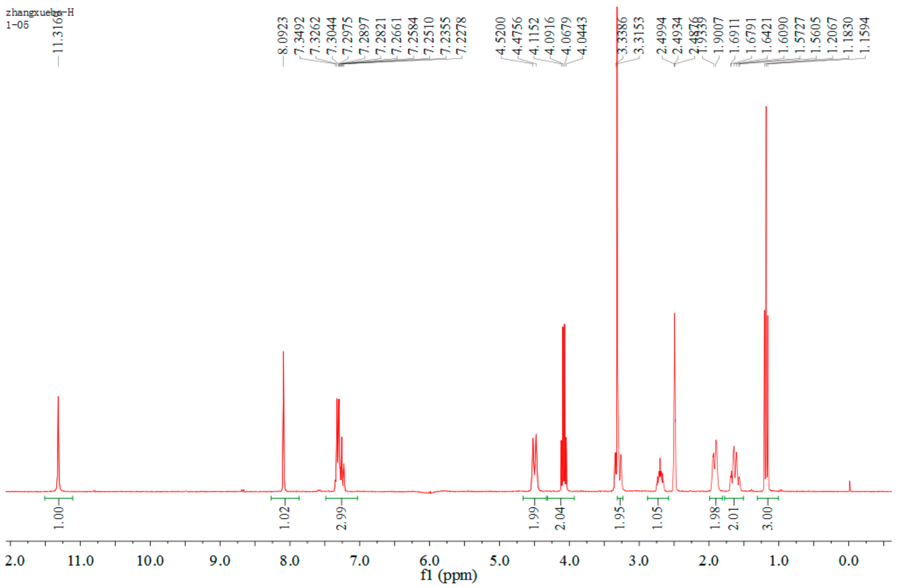

**Figure S40.** $^{1}H$ NMR spectra of the title compound **Ethyl (*E*)-1-(2-(3,5-difluorobenzylidene) hydrazine-1-carbonothioyl)piperidine-4-carboxylate (3s)**

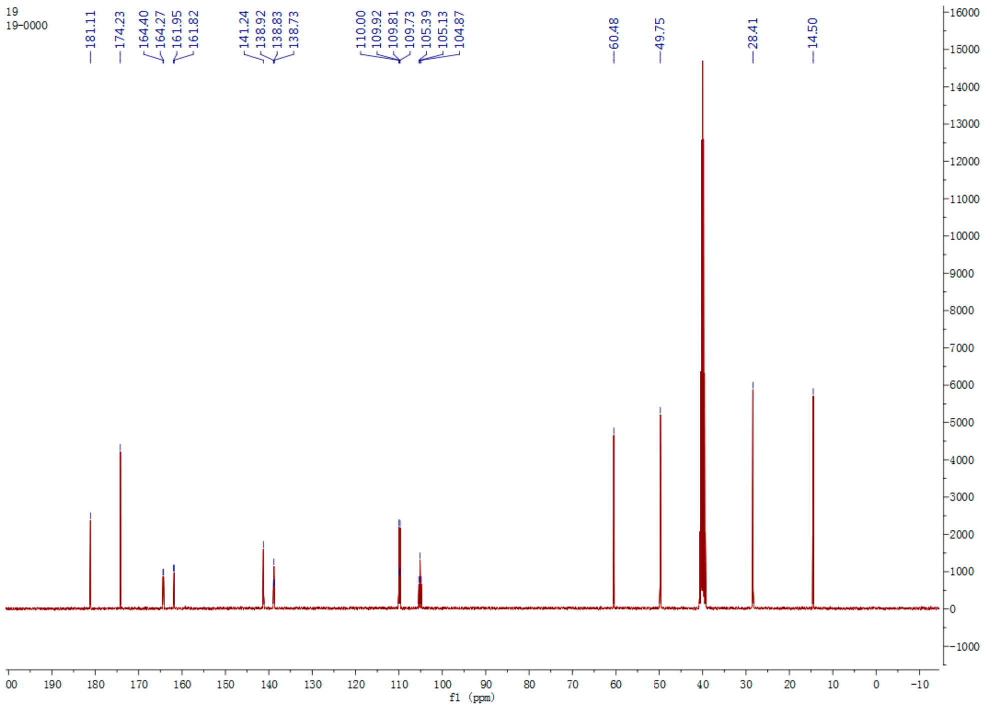

**Figure S41.** $^{13}C$ NMR spectra of the title compound **Ethyl (*E*)-1-(2-(3,5-difluorobenzylidene) hydrazine-1-carbonothioyl)piperidine-4-carboxylate (3s)**

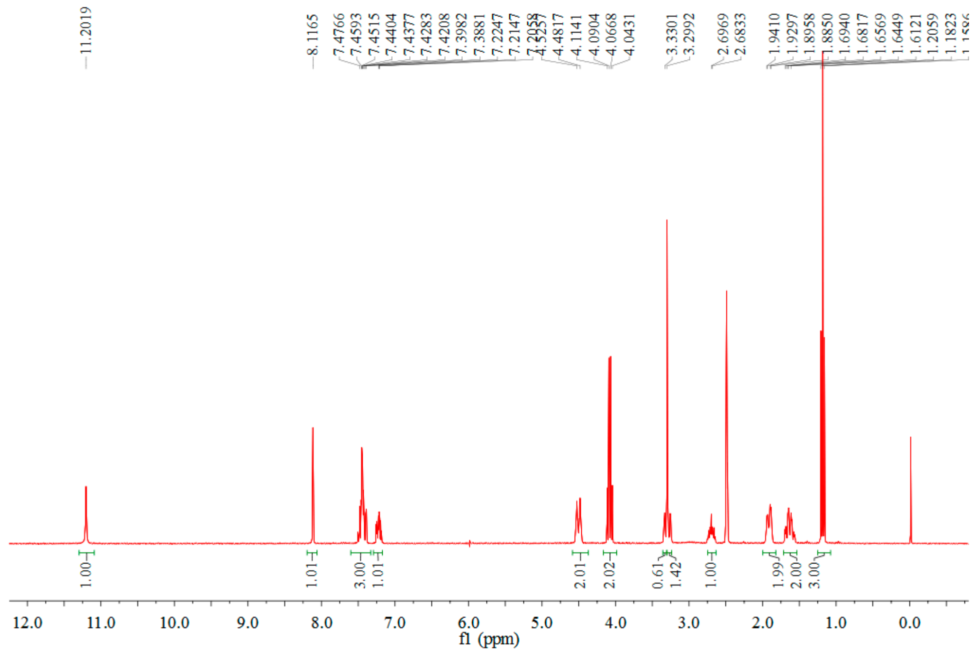


**Figure S42.** $^{1}H$ NMR spectra of the title compound **Ethyl (*E*)-1-(2-(3-fluorobenzylidene) hydrazine-1-carbonothioyl)piperidine-4-carboxylate (3t)**

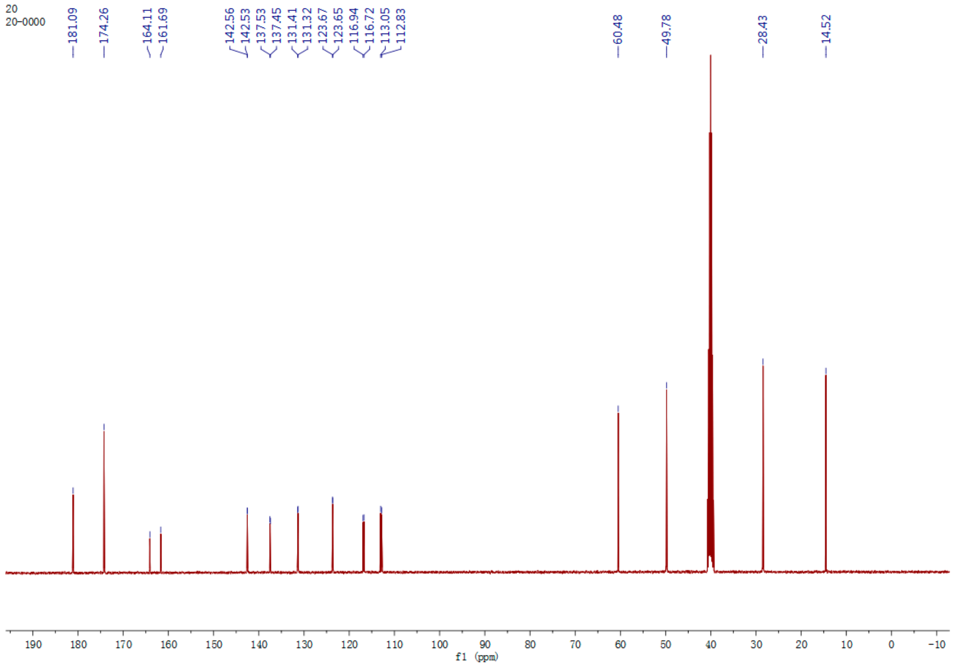


**Figure S43.** $^{13}C$ NMR spectra of the title compound **Ethyl (*E*)-1-(2-(3-fluorobenzylidene) hydrazine-1-carbonothioyl)piperidine-4-carboxylate (3t)**

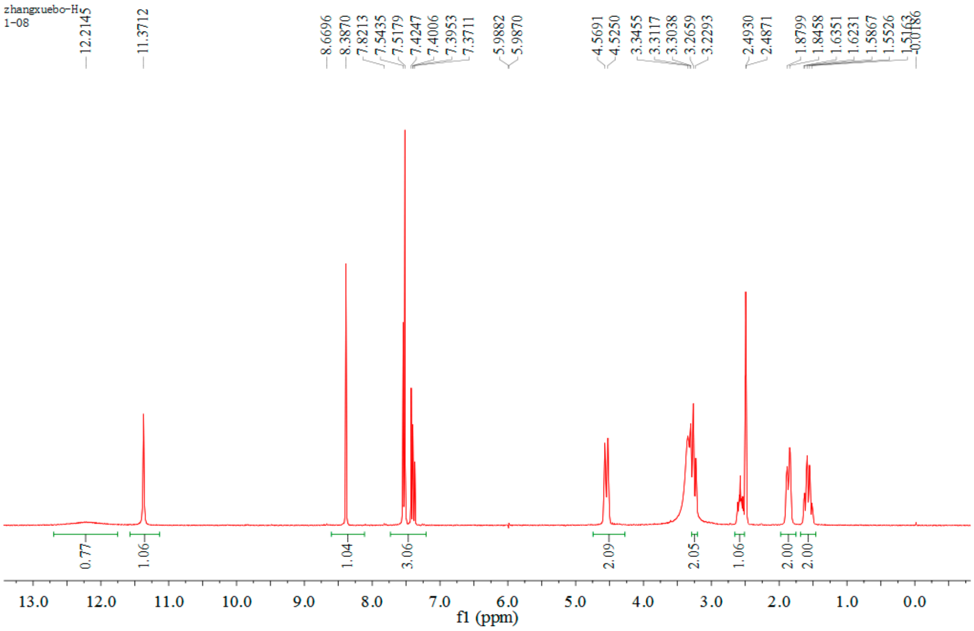


**Figure S44.** $^{1}H$ NMR spectra of the title compound **(*E*)-1-(2-(2,6-dichlorobenzylidene) hydrazine-1-carbonothioyl)piperidine-4-carboxylic acid (3u)**

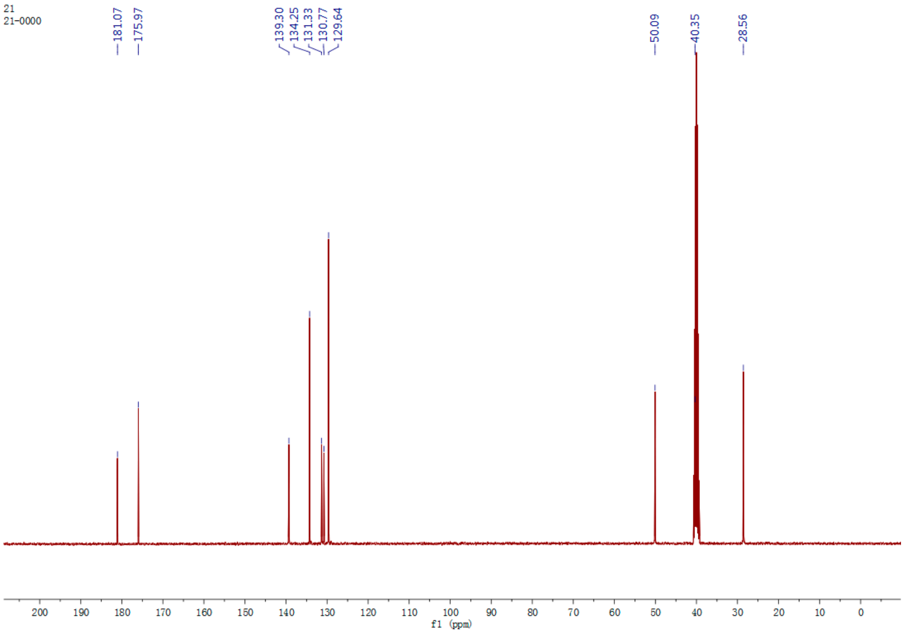


**Figure S45.** $^{13}C$ NMR spectra of the title compound **(*E*)-1-(2-(2,6-dichlorobenzylidene) hydrazine-1-carbonothioyl)piperidine-4-carboxylic acid (3u)**

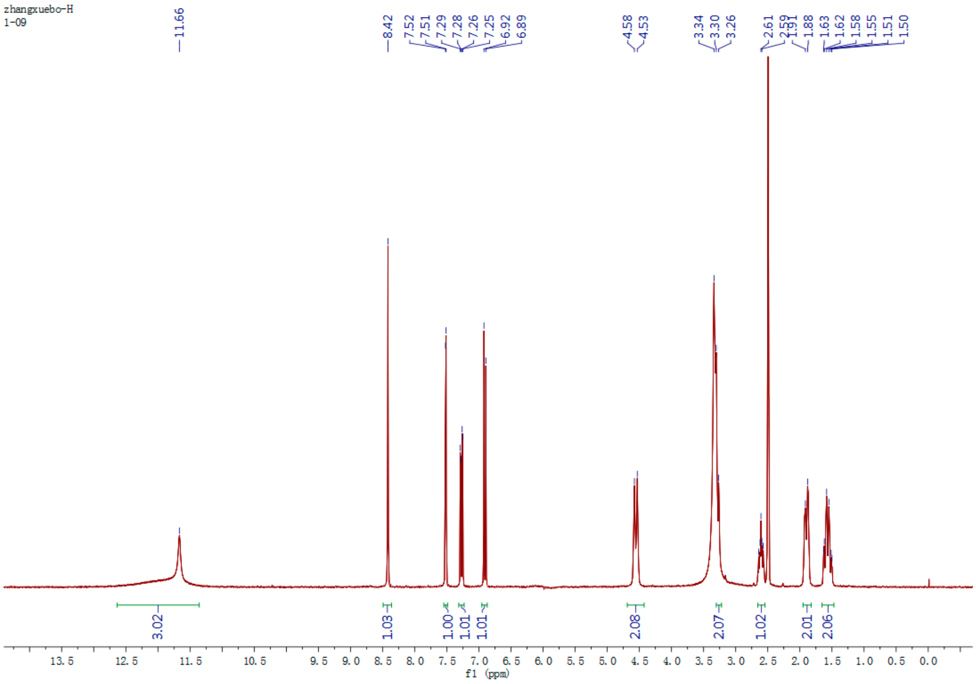


**Figure S46.** $^{1}$H NMR spectra of the title compound (*E*)-1-(2-(5-chloro-2-hydroxybenzylidene) hydrazine-1-carbonothioyl)piperidine-4-carboxylic acid (3v)

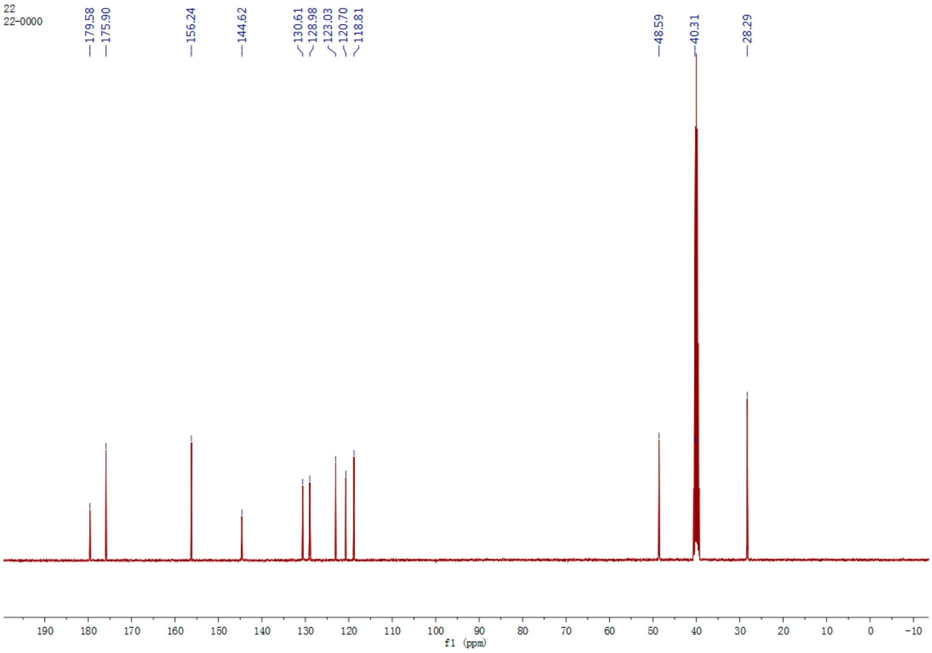


**Figure S47.** $^{13}$C NMR spectra of the title compound (*E*)-1-(2-(5-chloro-2-hydroxybenzylidene) hydrazine-1-carbonothioyl)piperidine-4-carboxylic acid (3v)

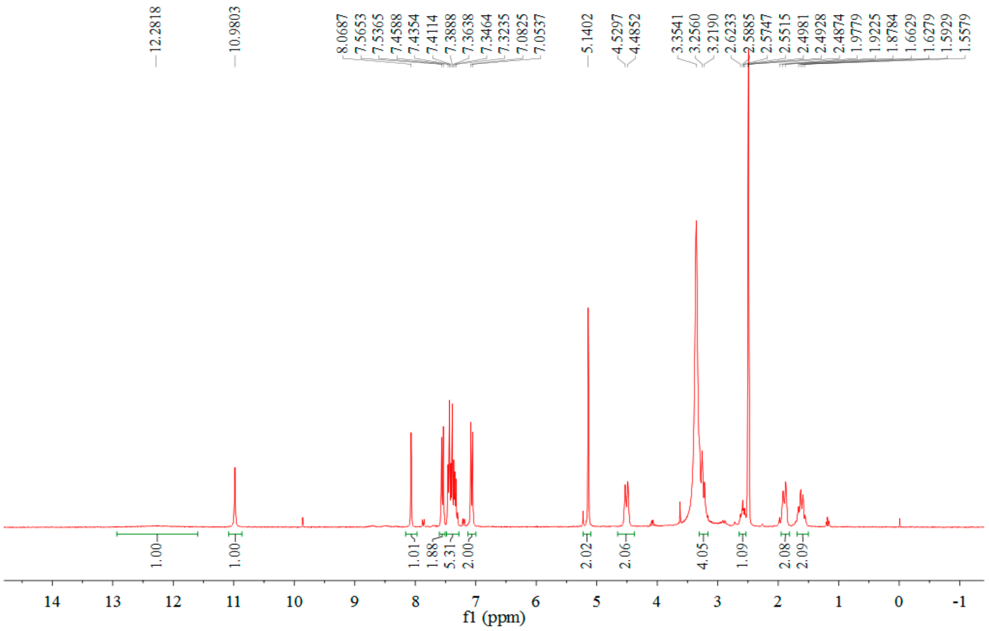


**Figure S48.** $^{1}H$ NMR spectra of the title compound **(*E*)-1-(2-(4-(benzyloxy)benzylidene) hydrazine-1-carbonothioyl)piperidine-4-carboxylic acid (3w)**

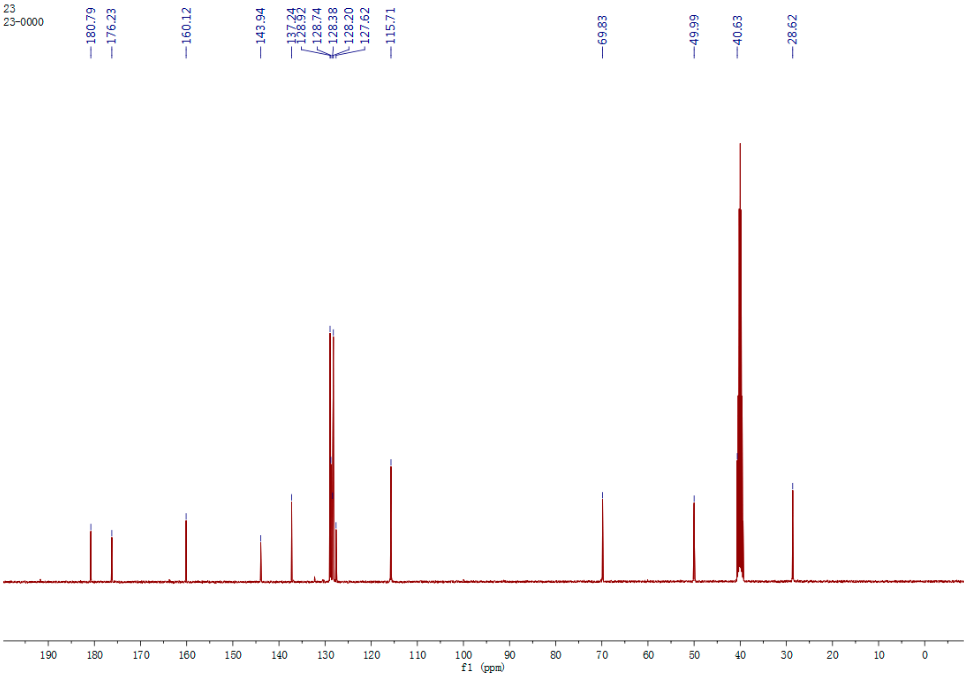


**Figure S49.** $^{13}C$ NMR spectra of the title compound **(*E*)-1-(2-(4-(benzyloxy)benzylidene) hydrazine-1-carbonothioyl)piperidine-4-carboxylic acid (3w)**

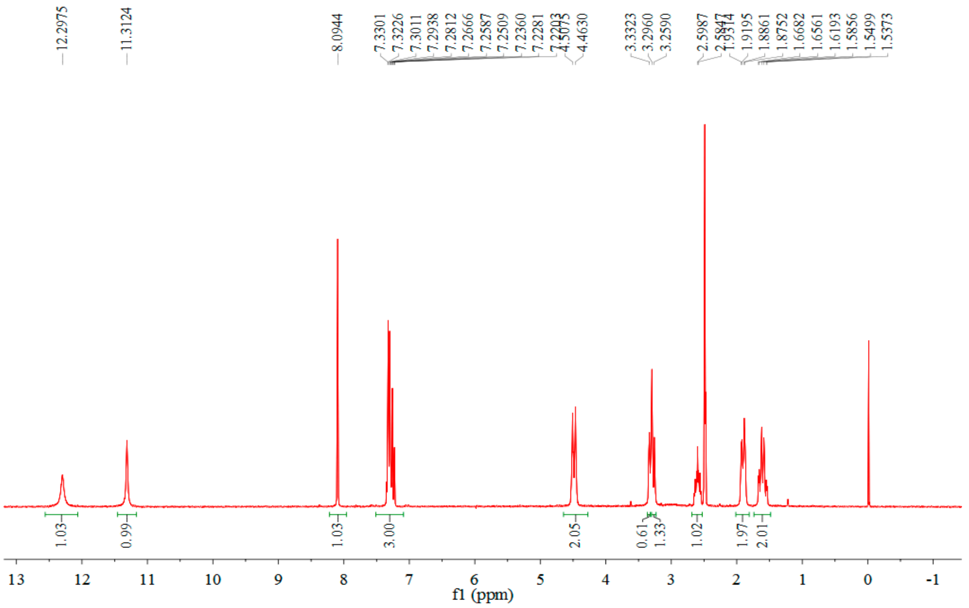


**Figure S50.** ¹H NMR spectra of the title compound (*E*)-1-(2-(3,5-difluorobenzylidene) hydrazine-1-carbonothioyl)piperidine-4-carboxylic acid (3x)

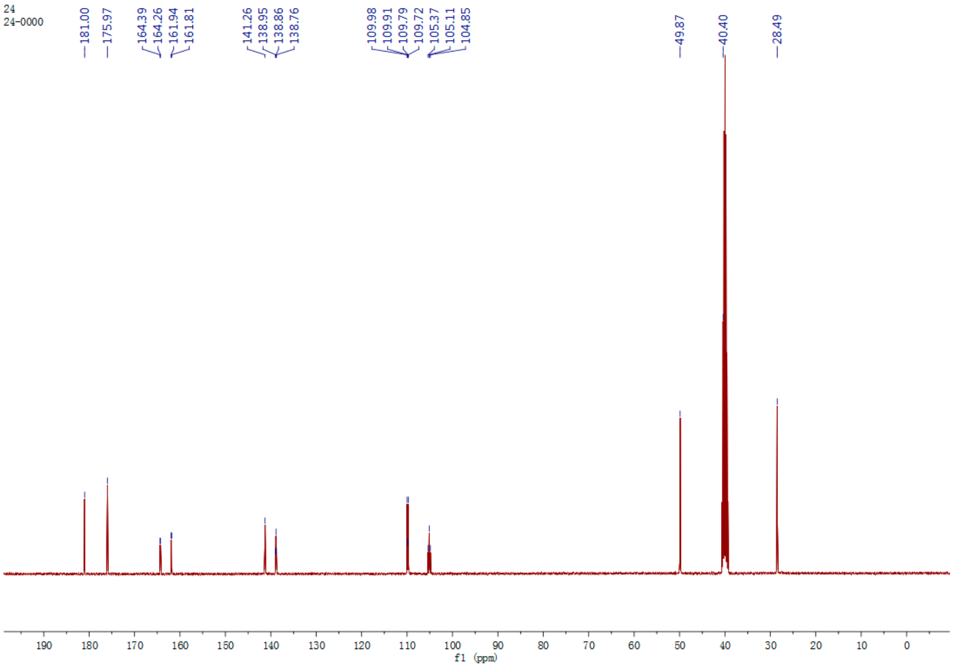


**Figure S51.** ¹³C NMR spectra of the title compound (*E*)-1-(2-(3,5-difluorobenzylidene) hydrazine-1-carbonothioyl)piperidine-4-carboxylic acid (3x)

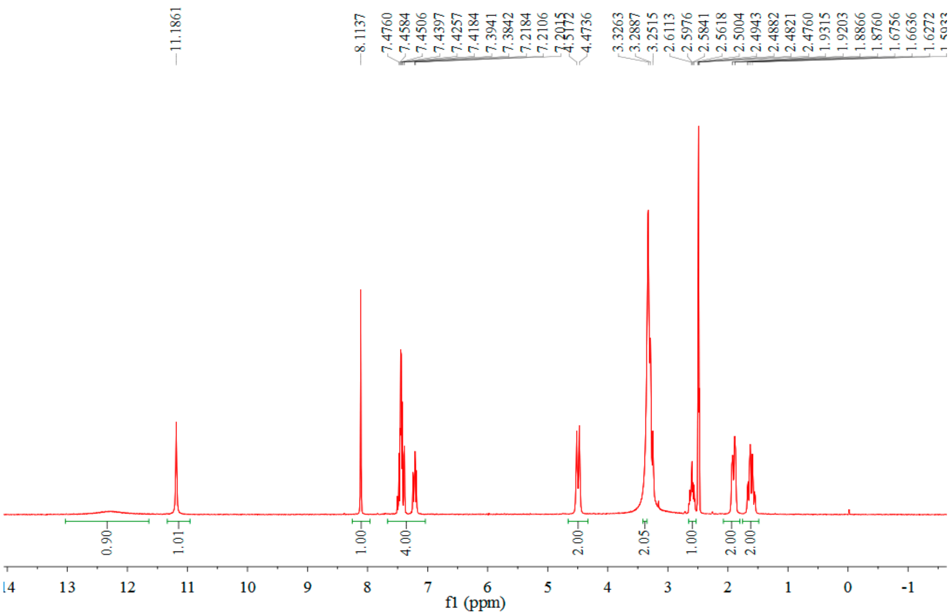


**Figure S52.** $^{1}$H NMR spectra of the title compound **(*E*)-1-(2-(3-fluorobenzylidene) hydrazine-1-carbonothioyl)piperidine-4-carboxylic acid (3y)**

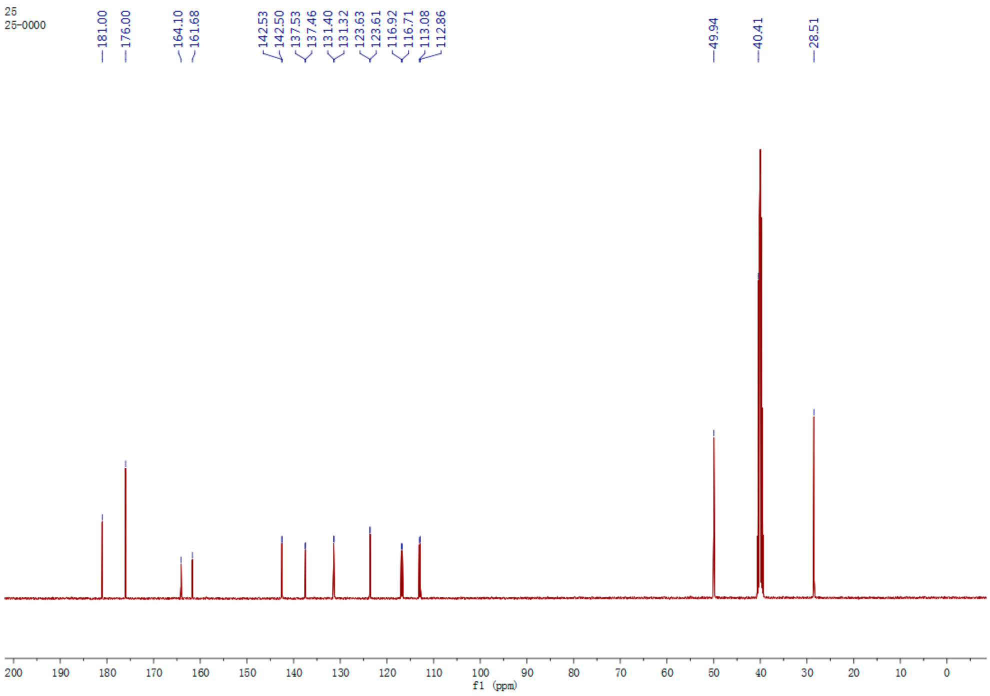


**Figure S53.** $^{13}$C NMR spectra of the title compound **(*E*)-1-(2-(3-fluorobenzylidene) hydrazine-1-carbonothioyl)piperidine-4-carboxylic acid (3y)**
